# Targeting SUMOylation with an injectable nanocomposite hydrogel to optimize radiofrequency ablation therapy for hepatocellular carcinoma

**DOI:** 10.1186/s12951-024-02579-1

**Published:** 2024-06-18

**Authors:** Junfeng Liu, Xi Li, Jiawen Chen, Jingpei Guo, Hui Guo, Xiaoting Zhang, Jinming Fan, Ke Zhang, Junjie Mao, Bin Zhou

**Affiliations:** 1 Center of Interventional Medicine, The Fifth Affiliated Hospital of Sun Yat-Sen University, Zhuhai, 519000 Guangdong Province China; 2 Institute of Interventional Radiology, Sun Yat-Sen University, Zhuhai, 519000 Guangdong Province China; 3 Center of Cerebrovascular Disease, The Fifth Affiliated Hospital of Sun Yat-Sen University, Zhuhai, 519000 Guangdong Province China; 4 Guangdong Provincial Engineering Research Center of Molecular Imaging, The Fifth Affiliated Hospital of Sun Yat-Sen University, Zhuhai, 519000 Guangdong Province China

**Keywords:** Hepatocellular carcinoma, Radiofrequency ablation, Small ubiquitin-like modifier 2, TAK-981, Nanocomposite hydrogel

## Abstract

**Background:**

Incomplete radiofrequency ablation (iRFA) in hepatocellular carcinoma (HCC) often leads to local recurrence and distant metastasis of the residual tumor. This is closely linked to the development of a tumor immunosuppressive environment (TIME). In this study, underlying mechanisms and potential therapeutic targets involved in the formation of TIME in residual tumors following iRFA were explored. Then, TAK-981-loaded nanocomposite hydrogel was constructed, and its therapeutic effects on residual tumors were investigated.

**Results:**

This study reveals that the upregulation of small ubiquitin-like modifier 2 (*Sumo2*) and activated SUMOylation is intricately tied to immunosuppression in residual tumors post-iRFA. Both knockdown of *Sumo2* and inhibiting SUMOylation with TAK-981 activate IFN-1 signaling in HCC cells, thereby promoting dendritic cell maturation. Herein, we propose an injectable PDLLA-PEG-PDLLA (PLEL) nanocomposite hydrogel which incorporates self-assembled TAK-981 and BSA nanoparticles for complementary localized treatment of residual tumor after iRFA. The sustained release of TAK-981 from this hydrogel curbs the expansion of residual tumors and notably stimulates the dendritic cell and cytotoxic lymphocyte-mediated antitumor immune response in residual tumors while maintaining biosafety. Furthermore, the treatment with TAK-981 nanocomposite hydrogel resulted in a widespread elevation in PD-L1 levels. Combining TAK-981 nanocomposite hydrogel with PD-L1 blockade therapy synergistically eradicates residual tumors and suppresses distant tumors.

**Conclusions:**

These findings underscore the potential of the TAK-981-based strategy as an effective therapy to enhance RFA therapy for HCC.

**Graphic Abstract:**

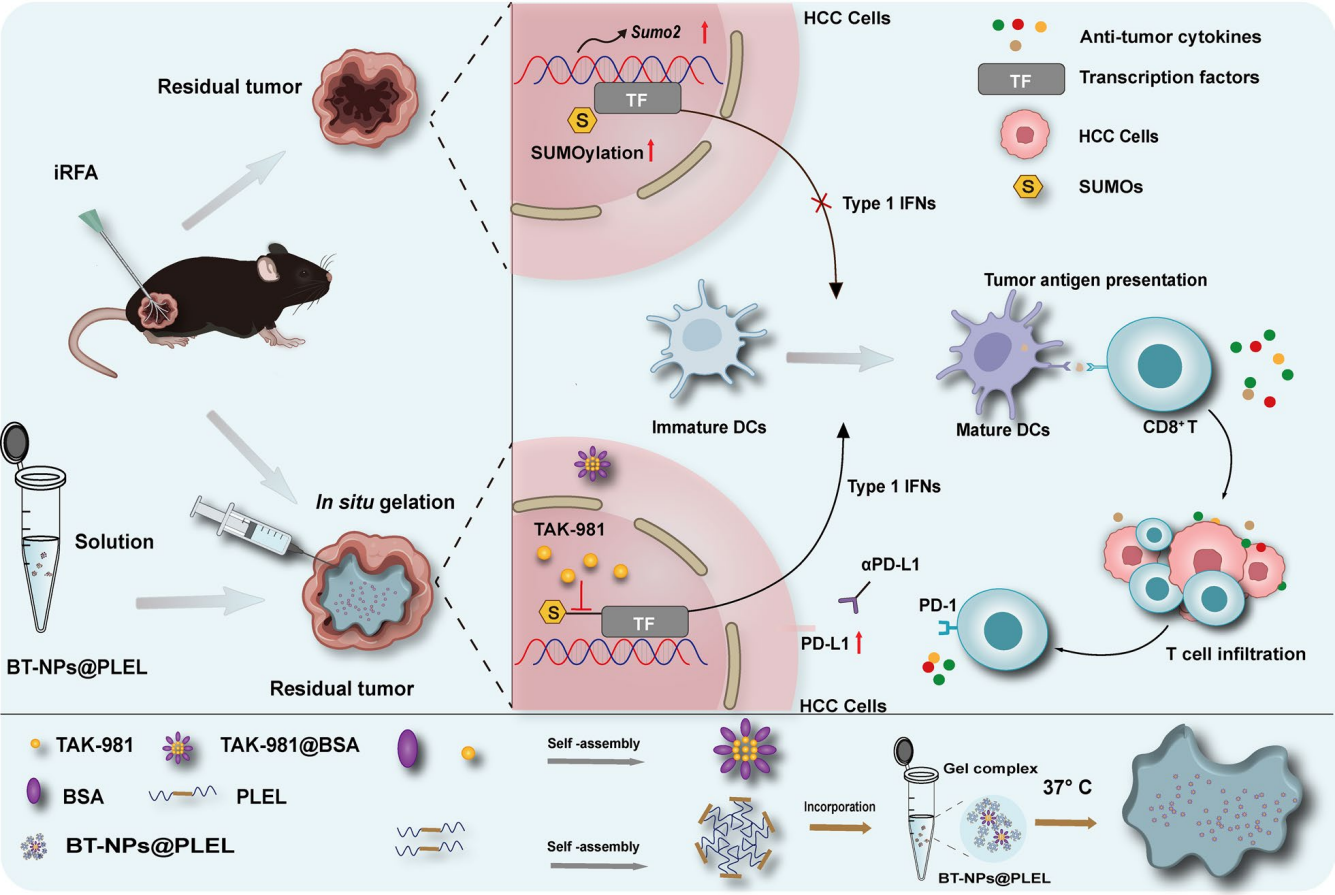

**Supplementary Information:**

The online version contains supplementary material available at 10.1186/s12951-024-02579-1.

## Introduction

Radiofrequency ablation (RFA) stands as a primary treatment for early-stage hepatocellular carcinoma (HCC), valued for its exceptional efficiency, minimal invasiveness, lower complication morbidity, and abbreviated hospital stays [1, 2]. However, RFA results are compromised by high rates of local relapse, which can reach up to 60% [3]. This recurrence is frequently attributed to the residual tumors that persist after incomplete RFA (iRFA) [4]. Additionally, the sublethal heat stimulation induced by iRFA has been observed to expedite the progression of these residual tumors. Current research indicates that this sublethal heat stimulation fosters a suppressive tumor immune microenvironment (TIME) and facilitates the survival and progression of the residual tumors [5–7]. However, the exact mechanisms driving these phenomena are indeed complex and still being explored.

The extensive tumor necrosis and profusion of cellular debris resulting from RFA might be expected to serve as tumor-specific antigens, potentially triggering an adaptive antitumor immune response [8]. However, clinical studies indicate that the immune response often falls short of completely eradicating or managing residual tumors in iRFA scenarios [9]. This insufficiency implies that antigen presentation, a crucial component of adaptive antitumor immunity, is impeded. In this process, dendritic cells (DCs) play a significant role in the antigen presentation process, promoting the infiltration and activation of cytotoxic T lymphocytes (CTLs) [10]. They specialize in antigen capture and processing, which are then presented to immunocompetent T cells. The Type I Interferon (IFN-1) signal notably impacts the activation, maturation, migration, and survival of DCs, and concurrently boosts the activity of CTLs [11]. However, the IFN-1/DCs/CTLs signal is frequently suppressed within the TIME due to various mechanisms [12]. Hence, inhibition of the IFN-1 pathway might contribute to the imbalanced immune response post-iRFA, though further research is imperative to understand this intricate mechanism comprehensively.

In this study, we revealed heightened small ubiquitin-like modifier protein 2 (*Sumo2*)-mediated SUMOylation activity in residual tumors after iRFA of HCC. SUMOylation is a post-translational modification process involving the attachment of SUMO proteins to substrates, is linked with increased tumorigenic potential and poorer clinical outcomes [13]. It has been reported to interfere with the IFN-1 signaling by modifying multiple targets. Inhibition of SUMOylation has shown promise in enhancing DC maturation and activating anti-tumor immune responses by stimulating IFN-1 production in diverse tumors during preclinical studies. Multiple ongoing or completed phase 1/2 clinical trials have evaluated the efficacy of small molecule SUMOylation inhibitors as potential immunotherapy [14, 15]. While no direct association exists between targeting SUMOylation and residual tumors after iRFA in existing studies, this novel approach shows promise for eliminating HCC residual tumors post-iRFA in future clinical applications. TAK-981, a novel small molecule inhibitor specifically designed to target the SUMOylation pathway with remarkable selectivity and potency [16, 17], is expected to be a compelling candidate for treating residual tumors after iRFA. However, SUMOylation is essential for preserving cell survival and functionality, and the systemic administration of SUMOylation inhibitors may result in potential toxicity [18–20]. TAK-981 has been primarily delivered via intratumoral injections in the case of solid tumors [21, 22]. However, intratumoral injections come with a set of limitations, including a restricted duration of drug retention and the occurrence of local reactions [23]. To achieve optimal efficacy, there is a risk of repeat administration and overexposure of TAK-981 due to the restricted duration of drug retention. This scenario may result in potential systemic and local toxicity stemming from overdosage and off-target delivery[23]. Therefore, it is of significant clinical importance to develop a novel drug formulation that can locally and sustainably release small molecule inhibitors, thereby enhancing anti-tumor efficacy while minimizing side effects.

*In-situ* forming hydrogels are viewed as potential carriers to address these challenges [24]. The biodegradable poly(d,L-lactide)-poly(ethylene glycol)-poly(d,L-lactide) (PDLLA-PEG-PDLLA; PLEL) triblock copolymer gel exhibits a flowable sol state at room temperature, which facilitates drug loading and injection. It undergoes phase transition to form a drug-loaded gel depot for sustained drug release upon in vivo administration. Moreover, PLEL hydrogels effectively prolong drug retention time [25, 26]. Nevertheless, the poor solubility of small-molecule drugs makes it difficult to achieve a uniform dispersion within hydrogels. Integrating nanomaterials into hydrogels achieves uniform drug dispersion and avoids drug burst release [27]. Bovine Serum Albumin (BSA) is widely used in drug delivery systems due to its beneficial properties, including easy drug loading, good water solubility, and non-toxicity. Its amphiphilic nature allows it to form nanoparticles with hydrophobic drugs and remain soluble in aqueous solutions [28]. In our study, BSA and TAK-981 first self-assembled to form nanoparticles, subsequently encapsulated within PLEL to obtain BT-NPs@PLEL nanocomposite hydrogels. The BT-NPs@PLEL was injected into residual tumor sites following iRFA, and the results indicate that BT-NPs@PLEL fostered the maturation of DCs, bolstered the infiltration and activation of CTLs, and effectively suppressed the residual tumors. Notably, extensive PD-L1 upregulation in residual tumors was observed following BT-NPs@PLEL treatment. The combination therapy of BT-NPs@PLEL and PD-L1 blockade effectively inhibits residual tumors and impedes the progression of distant tumors after iRFA. These findings underscore the potential of a TAK-981-based strategy in activating anti-tumor immunity, eradicating residual tumors, and optimizing RFA treatment.

## Result s

### Upregulation of *Sumo2* and activation of SUMOylation in residual tumors after iRFA

Research has consistently demonstrated that iRFA is associated with a rapid progression of residual tumors, and immunosuppression potentially plays a significant role. However, the source of immunosuppression is still unclear. To investigate this, we developed iRFA mouse models with Hepa1-6 cells, as shown in Figure S1A. Following a 21-day observation period after the iRFA treatment, the tumor growth curve indicates that the tumor volume in the iRFA-treated group escalated more rapidly compared to the untreated group (Figure S1B-C). The tumors were dissected and weighed, revealing a higher weight in the iRFA-treated group (Figure S1D). Subsequently, the TIME of the residual tumors was investigated. On day 21 following the iRFA treatment, tumor tissue was collected for flow cytometry analysis. The results indicate that while the proportions of total immune cells and DCs were not different across the two groups, there was a marked decrease in the infiltration of mature DCs within the iRFA-treated group (Figure S2A-C). DCs are essential for antigen presentation and stimulating anti-tumor immunity. Correspondingly, the infiltration of CD8^+^ T cells was also decreased in the iRFA-treated group (Figure S2D). These results suggest that iRFA may impede antigen presentation in residual tumors.

**Fig. 1:**
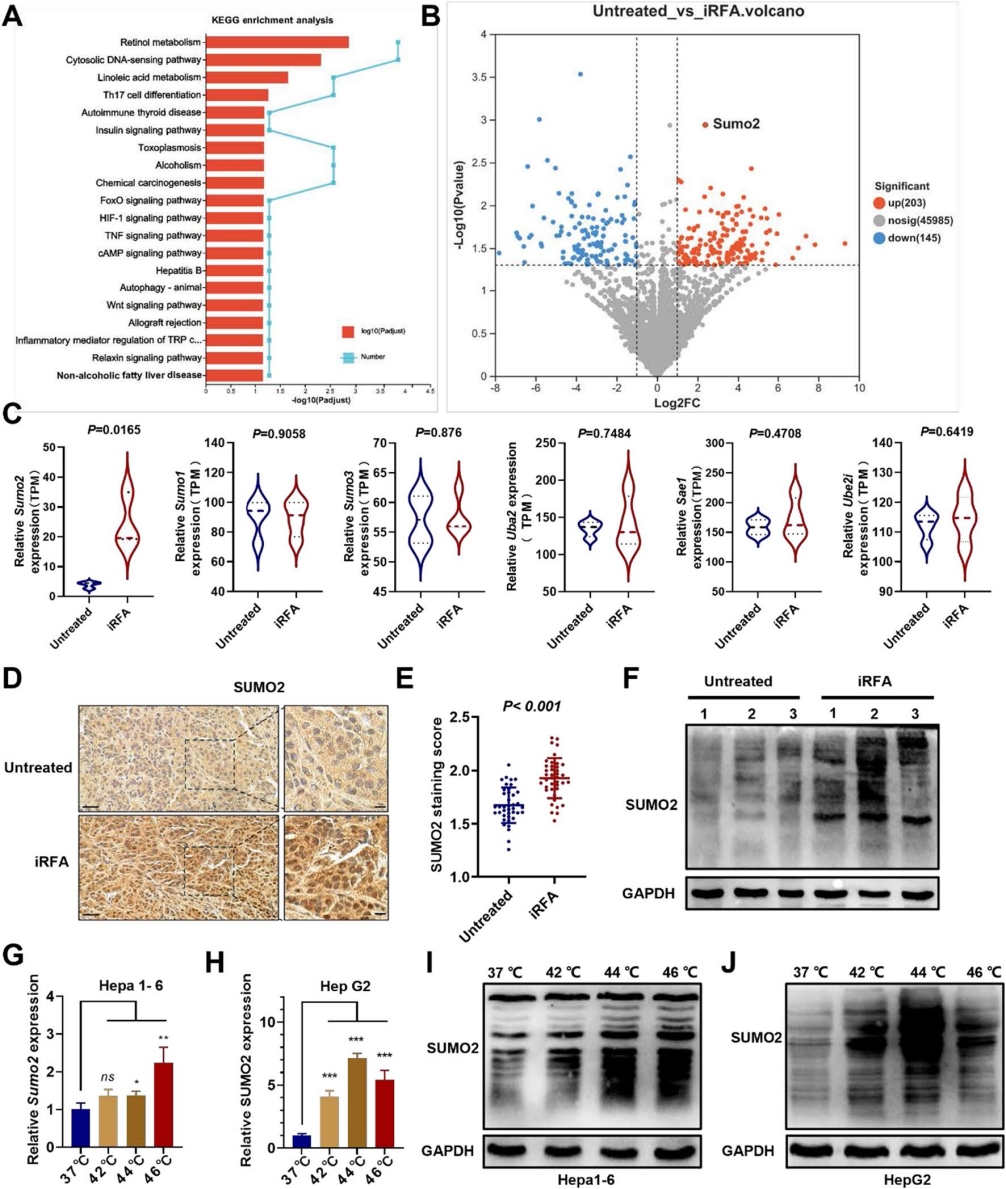
Upregulation of *Sumo2* and activation of SUMOylation in residual tumors after iRFA. **A** Significant enrichment of the DEGs of iRFA and untreated group in KEEG terms (top 20). **B** Volcano plot of DEGs in RNA-seq dataset. **C** Differences in expressions critical regulators in SUMOylation pathway between iRFA and the untreated group (n = 3). **D** The IHC staining of SUMO2 in tumor tissues, scale bar = 20 μm. **E** The IHC staining score of SUMO2 (n = 40). **F** Western blot analysis of conjugated-SUMO2 in tumor tissues. **G** The RT-qPCR analysis of the *Sumo2* expression in heated Hepa1-6 cells (n=3). **H** The RT-qPCR analysis of the *SUMO2* expression in heated HepG2 cells (n=3). **I** Western blot analysis of conjugated-SUMO2 in heated Hepa1-6 cells. **J** Western blot analysis of conjugated-SUMO2 in heated HepG2 cells. ns, not significant, *p < 0.05, **p < 0.01, ***p < 0.001

Suppression of antigen presentation could disrupt the anti-tumor immune response, potentially enabling residual tumors to persist and progress. To investigate the underlying mechanism, High-throughput transcriptome sequencing (RNA-Seq) was conducted on iRFA-derived residual tumor tissue. The pathway enrichment analysis shows that the cytosolic DNA-sensing pathway is a top enriched term (Fig. 1A). The cytosolic DNA-sensing pathway has been empirically linked to antigen presentation-dependent anti-tumor immunity, predominantly via the synthesis of IFN-1[29]. Consequently, we examined the downstream interferon-stimulated genes (ISGs) of IFN-1 signaling and discovered that the majority of ISGs were expressed at lower levels in the iRFA-treated group (Figure S3). The IFN-1 signal is crucial for promoting antigen presentation and DC activation. Suppression of IFN-1 signaling was considered responsible for inhibiting antigen presentation in residual tumors. Moreover, the differentially expressed genes (DEGs) analysis revealed that *Sumo2* was significantly up-regulated in residual tumor following iRFA in HCC (Fig. 1B, C, Figure S4). The expression levels of the SUMO pathway’s key components-*Sumo1*, *Sumo3*, *Uba2*, *Sae1*, and *Ube2i*-showed no significant difference between the iRFA-treated group and the untreated group (Fig. 1C). These findings are consistent with another iRFA-related dataset (GSE138224) in the GEO sequencing database (Figure S5) [5]. According to the TCGA database, lower *SUMO2* expression correlates with longer overall survival in HCC patients (Figure S6). The upregulation of SUMO2 protein was also observed in the residual tumor tissue with IHC staining (Fig. 1D, E), and an increased level of conjugated-SUMO2 was observed in the Western blot test (Fig. 1F). In line with these findings, in vitro simulation of iRFA using sublethal heat stimulation resulted in upregulated *Sumo2* expression and increased conjugated-SUMO2 in mouse Hepa1-6 HCC cells (Fig. 1G, I). A similar trend of upregulated SUMO2 expression and increased conjugated-SUMO2 was noted in human-derived HCC cells (HepG2 and Hep3B) following exposure to sublethal heat stress (Fig. 1H, J, Figure S7). SUMOylation has been reported to down-regulate the IFN-1 signaling [30]. ELISA testing of IFN-1 in tumor tissue also revealed impaired IFN-1 secretion following iRFA treatment (Figure S8). These results indicate that the sublethal heat stimulation induced by iRFA may inhibit the IFN-1 signal in HCC by activating *SUMO2*-mediated SUMOylation, potentially facilitating the development of the immunosuppressive microenvironment.

### Inhibiting the SUMO2-mediated SUMOylation activates the IFN-1 signal in iRFA HCC cells and promotes BMDCs maturation in vitro

**Fig. 2:**
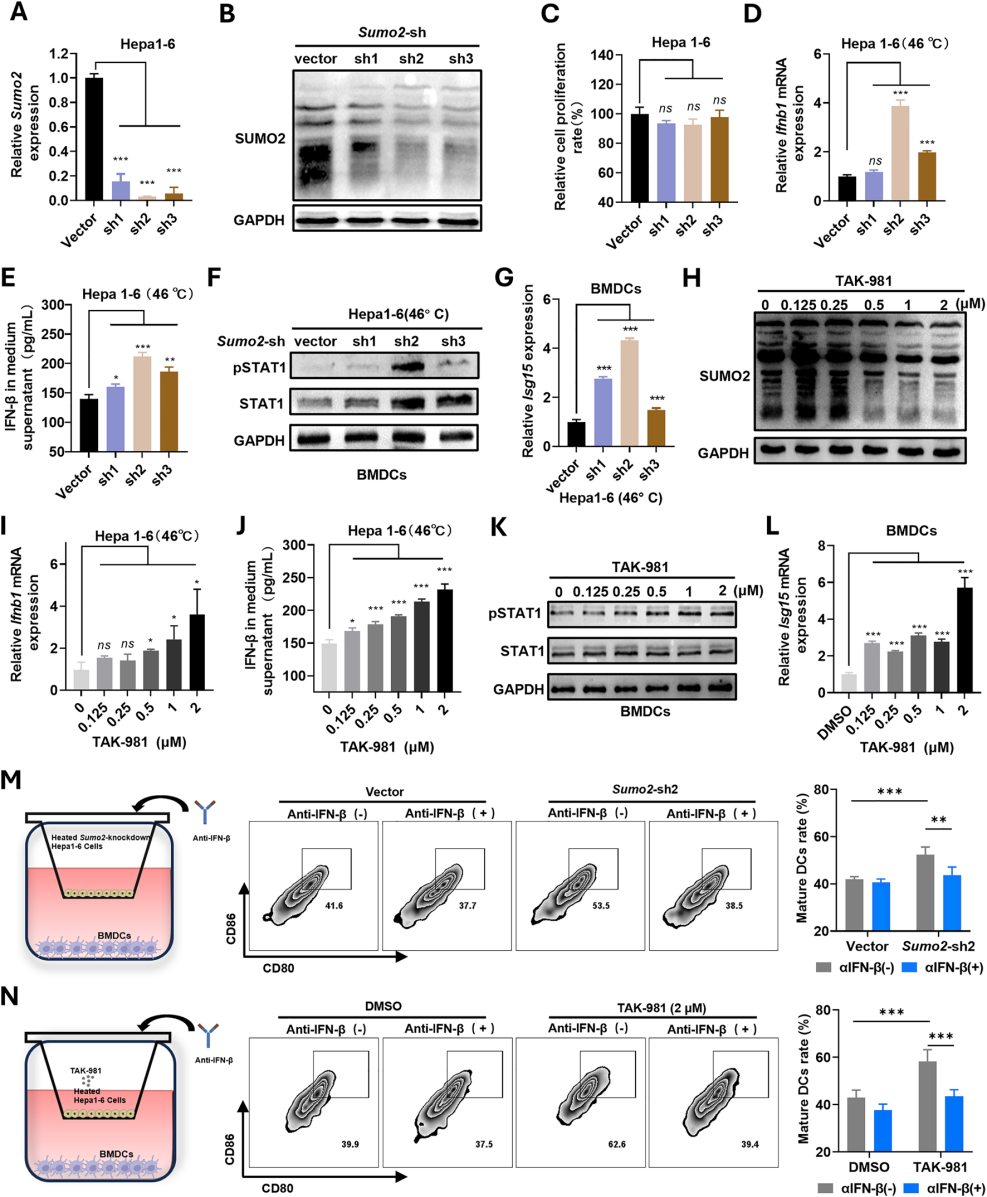
Knockdown of *Sumo2* or inhibition of SUMOylation effectively activates the IFN-1 pathway. **A** The RT-qPCR analysis of *Sumo2* in *Sumo2*-knockdown Hepa1-6 cells (n=3). **B** Western blot analysis of conjugated- SUMO2 in *Sumo2*-knockdown Hepa1-6 cells. **C** The relative proliferation rate in *Sumo2-*knockdown Hepa1-6 cells (n=3). **D** The RT-qPCR analysis of the *Ifnb1* gene in heated *Sumo2-*knockdown Hepa1-6 cells (n=3). **E** ELISA analysis of the IFN-β in the medium supernatant of heated *Sumo2*-knockdown Hepa1-6 cells (n=3). **F** Western blot analysis of STAT1/pSTAT1 in BMDCs co-cultured with heated Hepa1-6 cells. **G** The relative expression of *Isg15* in BMDCs co-cultured with heated Hepa1-6 cells (n=3). **H** Western blot analysis of conjugated-SUMO2 in Hepa1-6 cells treated with TAK-981. **I** The RT-qPCR analysis of the *Ifnb1* gene in Hepa1-6 cells treated with TAK-981 (n=3). **J** ELISA analysis of the IFN-β in the medium supernatant of Hepa1-6 cells treated with TAK-981 (n=3). **K** Western blot analysis of STAT1/pSTAT1 in BMDCs co-cultured with heated Hepa1-6 cells treated with TAK-981. **L** The relative expression of *Isg15* in BMDCs co-cultured with heated Hepa1-6 cells treated with TAK-981 (n=3). **M** The representative flow cytometry plots and statistical analysis of mature DCs rate after co-cultured with heated *Sumo2*-knockdown Hepa1-6 cells (n=6). **N** The representative flow cytometry plots and statistical analysis of mature DCs rate after co-cultured with heated Hepa1-6 cells treated with TAK-981. ns, not significant (n=6), *p < 0.05, **p < 0.01, ***p < 0.001

SUMOylation has previously been identified as suppressing the IFN-1 signal by modifying several targets within the cell [30]. In this study, we further investigated if knocking down *Sumo2* and inhibiting SUMOylation could stimulate the IFN-1 signal in HCC cells. To this end, we genetically engineered Hepa1-6 cells to interfere with *Sumo2* using short hairpin RNA (shRNA) (Fig. 2A). Following the knockdown of *Sumo2,* we observed a decrease in conjugated-SUMO2 in Hepa1-6 cells (Fig. 2B), while the cell proliferation rate was not significantly affected (Fig. 2C). Meanwhile, significantly higher *Ifnb1* expression and IFN-β secretion were observed in the *Sumo2*-knockdown heated HCC cells compared to the control group (Fig. 2D, E). Building on the understanding that Signal Transducer and Activator of Transcription 1 (STAT1) plays a pivotal role in the maturation and differentiation of DCs, we observed activation of STAT1 in Bone Marrow-Derived Dendritic Cells (BMDCs). This occurred following their co-culture with Hepa1-6 cells, in which *Sumo2* had been knocked down and subsequently heated (Fig. 2F). Furthermore, ISGs, exemplified by *Isg15*, were significantly upregulated in BMDCs following the same co-culture process (Fig. 2G, Figure S9). These results underscore the response of BMDCs to signals from interferons. Then the heated Hepa1-6 cells were cultured with an increased concentration of TAK-981, and we observed a gradual reduction in the conjugated-SUMO2. Concurrently, there was an elevation in the expression of *Ifnb1* and secretion of IFN-β in these heated Hepa1-6 cells (Fig. 2H, I, J). Similarly, STAT1 was activated (Fig. 2K), and ISGs were significantly up-regulated in BMDCs when co-culturing with heated Hepa1-6 cells treated with TAK-981 sequentially (Fig. 2L, Figure S10). These findings provide strong evidence of a negative correlation between SUMO2-mediated SUMOylation and the IFN-1 signaling pathway in HCC cells. They also demonstrate that BMDCs could be affected by the IFN-1 secreted by HCC cells when co-cultured together. Therefore, this co-culture system of HCC cells and BMDCs was further utilized to simulate the in vivo TIME of the iRFA tumors, and the flow cytometry analysis showed that both knockdown *Sumo2* and treating Hepa1-6 cells with TAK-981 resulted in increased proportions of matured DCs. However, when IFN-β was neutralized with an antibody, this “pro-maturation effect” was reversed (Fig. 2M, N). These results emphasize the potential of targeting SUMO2-mediated SUMOylation as a treatment approach to enhance antigen presentation in residual tumors after iRFA.

### Preparation and characterization of BT-NPs@PLEL

**Fig. 3:**
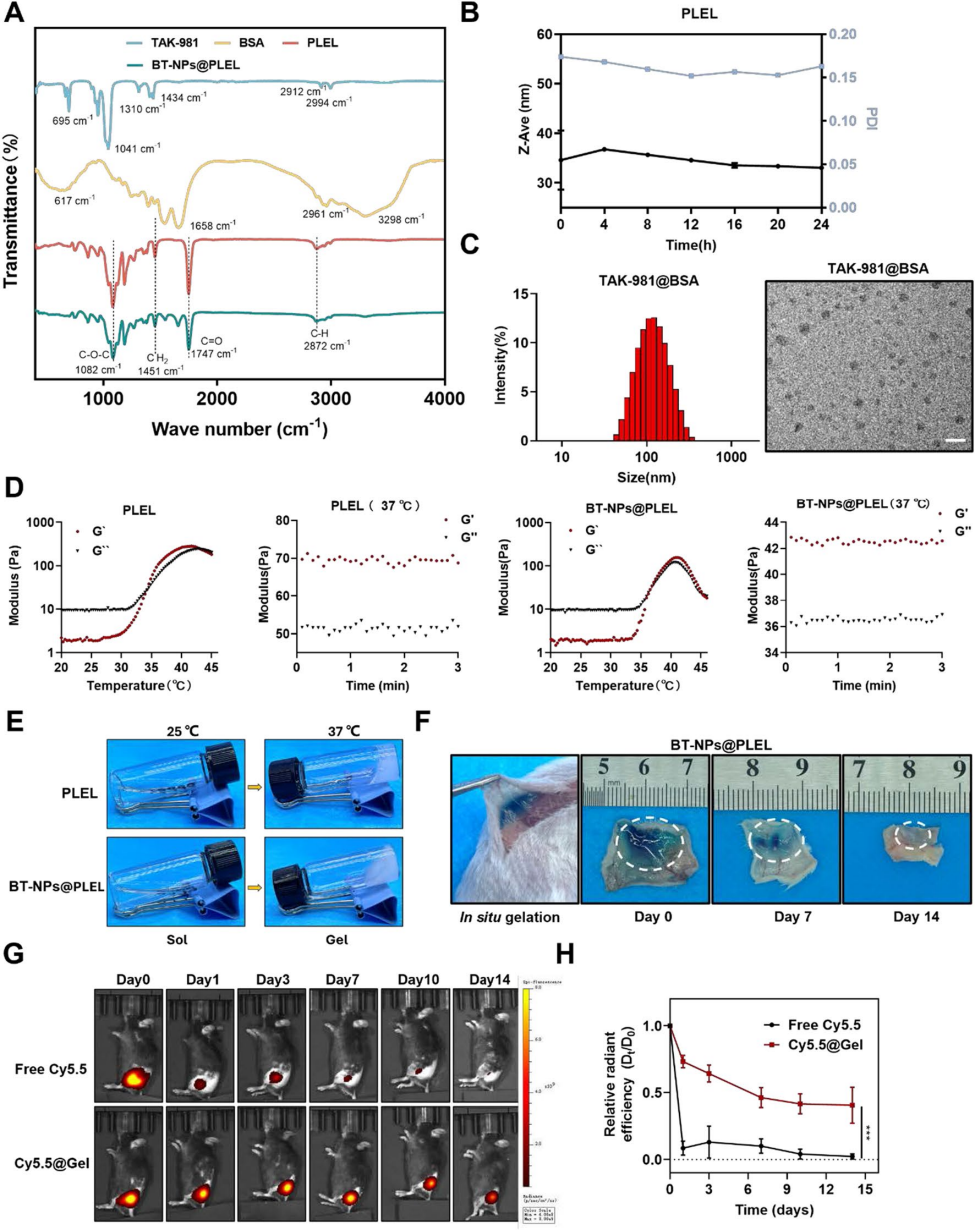
Preparation and Characterization of BT-NPs@PLEL. **A** FTIR spectra analysis of TAK-981, BSA, PLEL, and BT-NPs@PLEL. **B** The hydrodynamic diameter and polymer dispersity index of PLEL-Sol (1 wt%) within 24 h. **C** The hydrodynamic diameter and the TEM image of TAK-981@BSA nanoparticles, scale bar = 200 nm. **D** The rheological behavior of PLEL (25 wt%), BT-NPs@PLEL (25 wt%) in dependent of temperature. G′, storage modulus; G″, loss modulus. **E** Photographs showing the macroscopic thermo-sensitive sol–gel translation of PLEL and BT-NPs@PLEL (25 wt%). **F** In vivo gelation and degradation behavior of BT-NPs@PLEL (25 wt%) at different time points. **G**, **H** IVIS images and statistical analysis of fluorescence signal recorded at different times after injection of Cy5.5 and Cy5.5@Gel (n=3). ***p < 0.001

To enhance the therapeutic efficacy of TAK-981 in treating residual tumors post-iRFA, we engineered a nanocomposite thermosensitive hydrogel known as BT-NPs@PLEL. The synthesized PLEL block compounds were first analyzed by Fourier transform infrared spectroscopy (FTIR). In the FTIR spectrum (Fig. 3A), a strong C = O stretching band appeared at 1747 cm^−1^, attributed to the ester carbonyl bond. The absorption band at 1082 cm^−1^ corresponds to the C–O–C asymmetric stretching vibration of the ether group in PEG. The peak at 2872 cm^−1^ and 1451 cm^−1^ correspond to the –CH_2_ asymmetric stretching vibration of both PDLLA and PEG, consistent with previous reports [31]. Meanwhile, the hydrodynamic size of triblock copolymer PLEL micelle solution (1 wt%) at 25 °C was measured to be about 25 nm (Figure S11), which remained nearly unchanged at room temperature over 24 h (Fig. 3B). BSA nanoparticles carrying TAK-981 were synthesized via the self-assembly method by hydrophilic and hydrophobic interactions. The transmission electron microscopy (TEM) revealed the uniform morphology of TAK-981@BSA, and the hydrodynamic size of TAK-981@BSA NPs was measured to be approximately 128nm (Fig. 3C). Subsequently, the TAK-981@BSA NPs were encapsulated into PLEL to construct the final composite gel (BT-NPs@PLEL). In addition, the peak of BT-NPs@PLEL in FTIR was consistent with PLEL (Fig. 3A), indicating that the introduction of TAK-981@BSA NPs did not change the chemical structure of PLEL. The Lower Critical Gelation Temperature (LCGT) of PLEL and BT-NPs@PLEL hydrogels is influenced by molecular weight and polymer concentration [26]. As PLEL concentration increases, the LCGT decreases (Figure S12). However, below 25 wt% PLEL, the LCGT of BT-NPs@PLEL exceeds body temperature, which makes it challenging to guarantee that the sol-gel transition occurs upon injection into the body temperature. Higher PLEL concentrations slow degradation, potentially causing inflammation [32]. Therefore, we selected a 25 wt% concentration for the nanocomposite hydrogels. To further strengthen the finding between embedded contents and gelling abilities, the temperature of sol–gel transition of PLEL and BT-NPs@PLEL was measured through the rheological behavior test. The sol–gel transition is defined as the point where the storage modulus (G′) exceeds the loss modulus (G′′). PLEL and BT-NPs@PLEL were present in the sol state at room temperature (25 °C) and turned into the gel state at body temperature (37° C) (Fig. 3D). Meanwhile, the macroscopic views of the gelation process of PLEL and BT-NPs@PLEL (25 wt%) are shown in Fig. 3E. Then, we examined the in vivo gelation and biodegradation behavior of BT-NPs@PLEL (mixed with the blue dye to be visualized). Gelation occurred swiftly following subcutaneous injection, most of which degraded within 14 days after the injection (Fig. 3F). In our subsequent experiments, we evaluated the effectiveness of drug delivery systems in facilitating the slow release of the drug. Remarkably, about 80% of the encapsulated TAK-981 could be released sustainably over 14 days in a PBS buffer at 37° C (Figure S13). Furthermore, free Cy5.5 (substitution for TAK-981) or Cy5.5@Gel (substitution for BT-NPs@PLEL) was injected subcutaneously and monitored by an in vivo fluorescence imaging system (PerkinElmer IVIS Lumina III) at different time points post-injection. It was found that the fluorescence signal from Cy5.5 rapidly diminished at the injection site injected with free Cy5.5, demonstrating that free drugs could diffuse quickly. In contrast, for the Cy5.5@Gel group, the fluorescence signal was maintained at high levels within tumors for 14 days (Fig. 3G, H), demonstrating the significantly prolonged retention of drugs with the help of the nanocomposite hydrogel.

### BT-NPs@PLEL activates anti-tumor immunity and suppresses residual tumors after iRFA

**Fig. 4:**
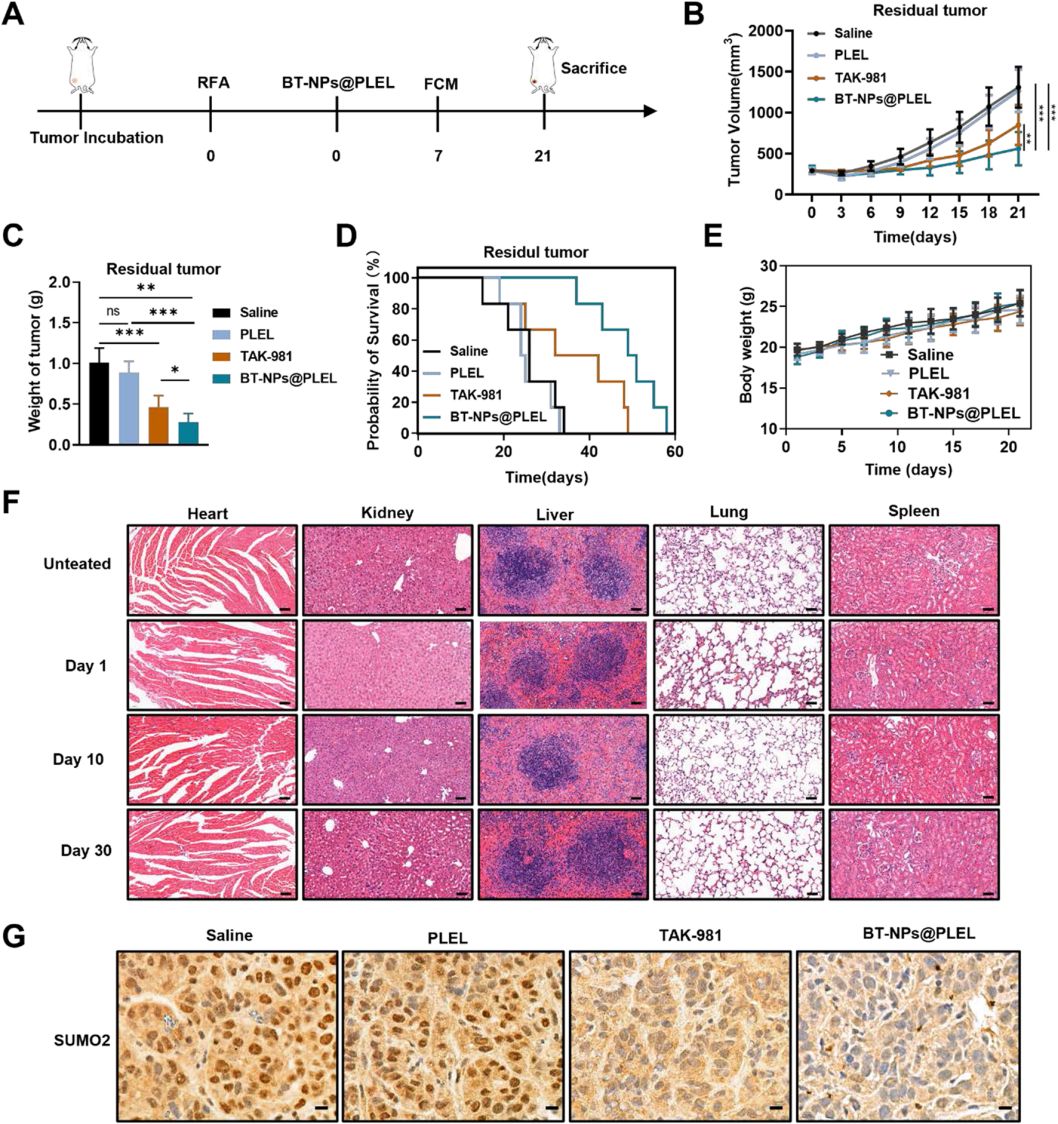
Inhibition of residual tumor after iRFA in vivo and blocking SUMOylation of the BT-NPs@PLEL*.* **A** Schematic representation of treatment of residual tumor after iRFA in C57BL/6 mice. **B** Tumor volume of residual tumors in different groups (n=6). **C** The weight of residual tumors post-iRFA on day 21 after different treatments (n=6). **D** Survival analysis of experimental mice in different groups (n=6). **E** The body weight changes of mice during treatment. **F** Images of H&E staining of tissue sections in essential organs after treatment with BT-NPs@PLEL on day 1, day 10, and day 30, scale bar: 50 µm. **G** Immunohistochemical analysis of SUMO2 in nucleus of residual tumor tissue after different treatments, scale bar: 10 µm. ns, not significant, *p < 0.05, **p < 0.01, ***p < 0.001

Driven by the promising in vitro results regarding the maturation of DCs through SUMOylation targeting and the prolonged retention of TAK-981 in BT-NPs@PLEL, the anti-tumor efficacy and the capacity to activate adaptive anti-tumor immunity were further investigated in iRFA mouse models. This investigation was conducted in accordance with the outlined therapeutic schedule (Fig. 4A). Hepa1-6-bearing mice were intratumorally injected with Saline, PLEL, TAK-981, and BT-NPs@PLEL after iRFA treatment. The tumor growth curve revealed that TAK-981 somewhat curtailed tumor growth when compared to the saline group, but no notable tumor suppression was seen in the PLEL groups. On the other hand, the residual tumor growth of BT-NPs@PLEL group was significantly inhibited (Fig. 4B). Additionally, an evaluation of the anti-tumor efficacy of BT-NPs@PLEL in residual tumors, excised from mice on day 21, revealed a significant reduction in both tumor volume and weight in the group treated with BT-NPs@PLEL, compared to other groups (Figure S14, Fig. 4C). In line with these results, the Kaplan–Meier survival curve indicates that the group treated with BT-NPs@PLEL exhibited a more extended survival period than the other groups (Fig. 4D). Crucially, no significant variations were observed in the body weight changes of the treated mice during the experiment (Fig. 4E). Besides, the serum biochemistry assay, complete blood panel test, and H&E staining of organ sections were performed, which further indicated no apparent systemic toxicity induced by BT-NPs@PLEL (Figure S15, Fig. 4F). These findings collectively suggest that BT-NPs@PLEL significantly improved the effectiveness of TAK-981 in treating iRFA residual tumor while ensuring safety. We also performed SUMOylation analysis on residual tumor tissues after treatment. Given that SUMOylation primarily occurs in the nucleus [17], we carried out immunohistochemical staining on the post-treatment residual tumor tissue. Our findings showed a notable decrease in SUMO2 staining within the nucleus of the residual cancer tissues after treatment (Fig. 4G). The results confirmed that BT-NPs@PLEL effectively blocked SUMOylation in residual tumor after iRFA.

**Fig. 5:**
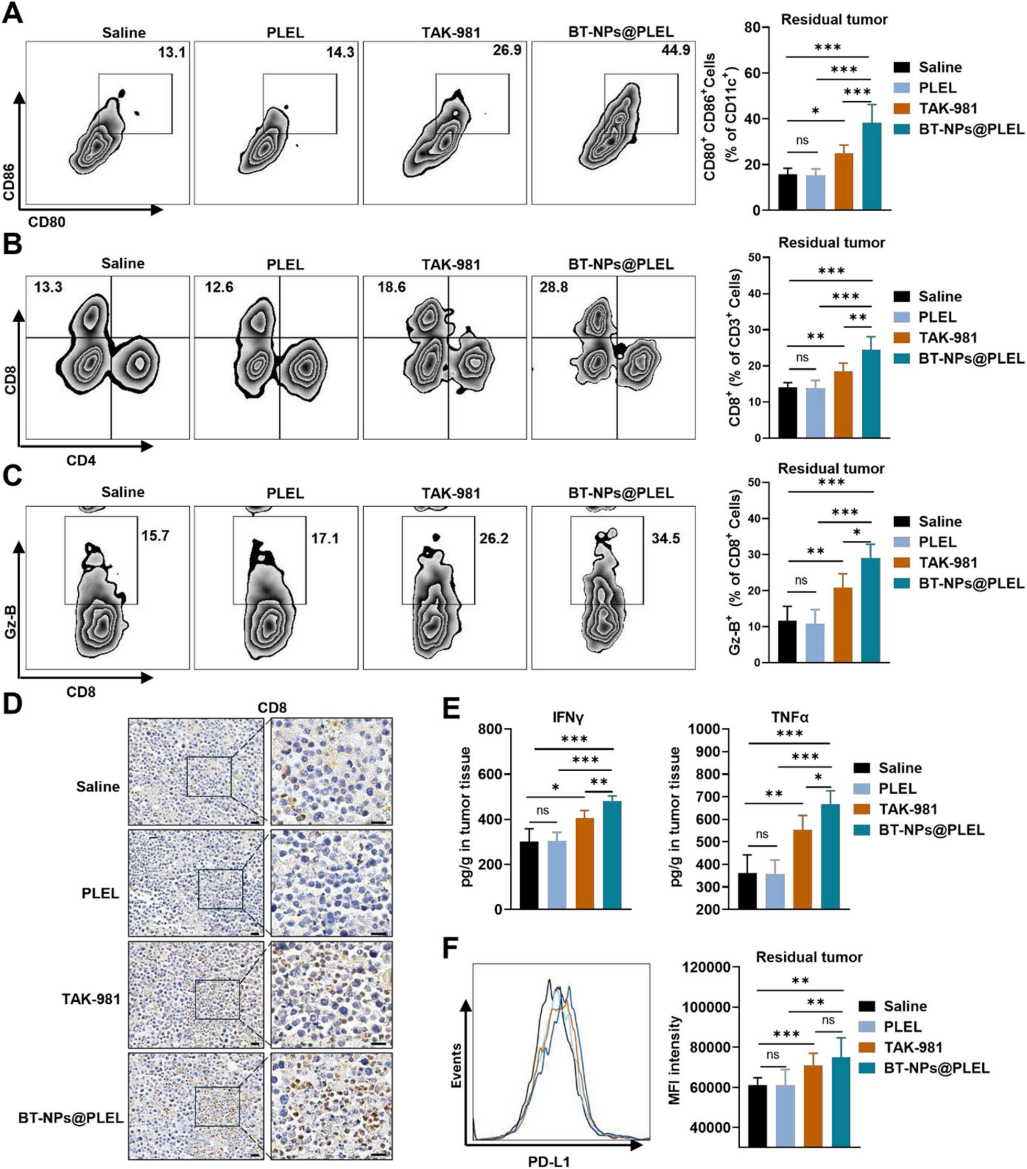
BT-NPs@PLEL activates anti-tumor immunity of residual tumor after iRFA. **A** Representative flow cytometry plots and proportions of mature DCs on day 7 (n = 6). **B** Representative flow cytometry plots and proportions of CD8^+^ T cells on day 7 (n = 6). **C** Representative flow cytometry plots and proportions of Granzyme B^+^ on day 7 (n = 6). **D** IHC staining of CD8 in residual tumor tissue, scale bar: 20 µm. **E** ELISA assay to detect the amount of IFN-γ and TNF-α in tumor tissue (n = 6). **F** The flow cytometry validation of PD-L1 overlay histogram and the mean fluorescence of the cells of tumor tissue after different treatments (n = 6). ns, not significant, *p < 0.05, **p < 0.01, ***p < 0.001

To shed light on the primary immune mechanisms contributing to the anti-tumor therapeutic efficacy observed in this study, we assessed adaptive anti-tumor immunity following various treatments. As expected, the proportions of mature DCs in the BT-NPs@PLEL group were significantly higher than in other groups (Fig. 5A). Correspondingly, there was a significant increase in the proportions of CD8^+^ T cells and their expression of Granzyme-B (Fig. 5B, C). Immunohistochemical staining also confirmed a substantial increase in CD8^+^ T cells infiltration following BT-NPs@PLEL treatment (Fig. 5D). TAK-981 treatment alone already showed considerable effectiveness in enhancing the infiltration of mature DCs and CD8^ +^ T cells, showing a greater effect than either the saline or PLEL groups. The BT-NPs@PLEL displayed an improved efficacy. Additionally, ELISA detection indicated that the group administered with BT-NPs@PLEL saw an increase in pro-inflammatory cytokines, IFN-γ and TNF-α, within the residual tumors. These were significantly higher than in the other groups (Fig. 5E). Nevertheless, it’s worth noting that earlier studies have shown a link between exposure to TAK-981 and a broad increase in PD-L1 within the TIME [21]. Consequently, we conducted an additional evaluation of PD-L1 expression and noted an increase in PD-L1 expression of the residual tumors following BT-NPs@PLEL treatment (Fig. 5F). This sets the stage for a combination of anti-PD-L1 therapy and BT-NPs@PLEL.

### Combining BT-NPs@PLEL with anti-PD-L1 treatment enhanced anti-tumor effects in residual tumors after iRFA

**Fig. 6:**
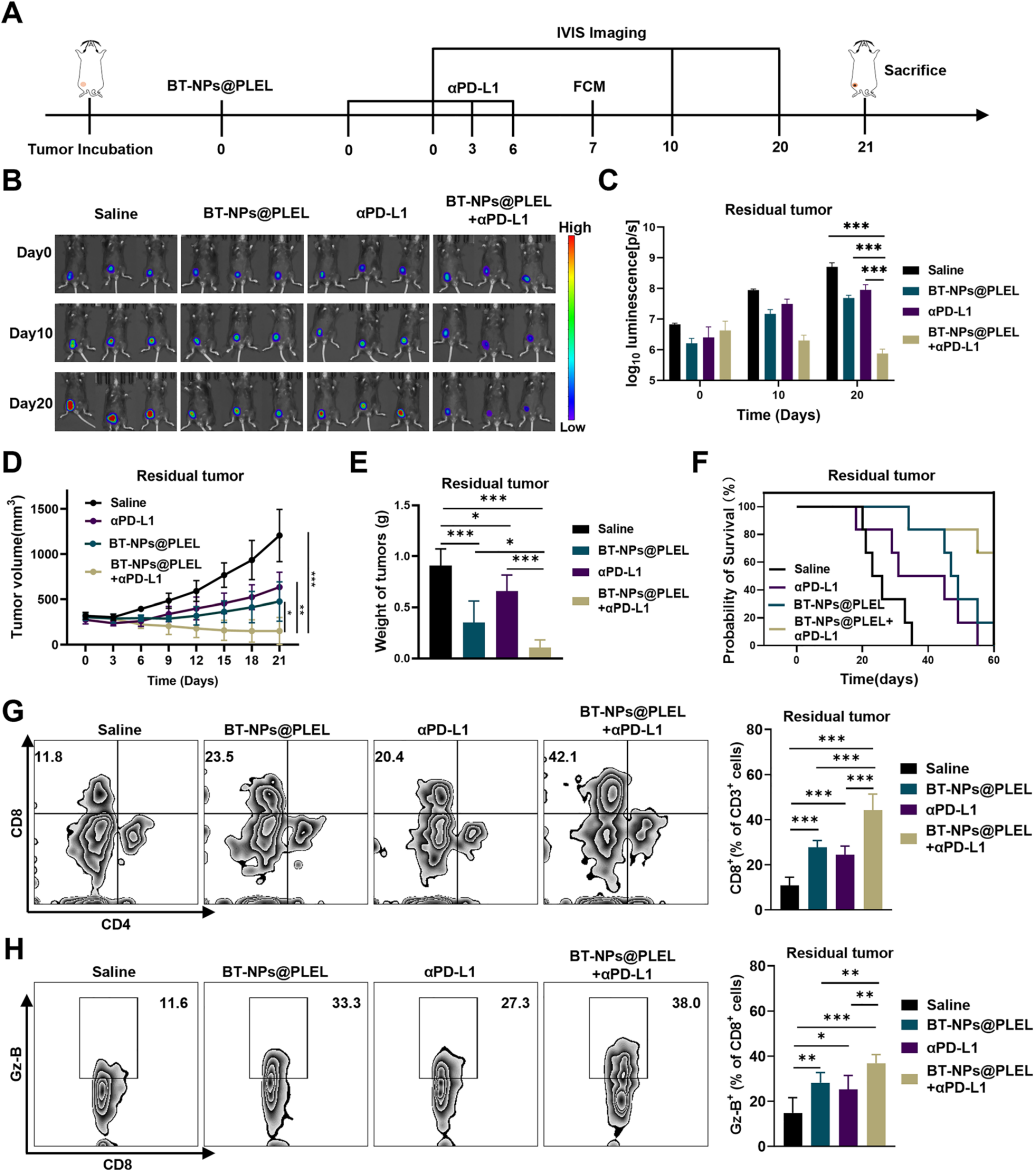
Combining BT-NPs@PLEL with anti-PD-L1 treatment for inhibition in residual tumors after iRFA. **A** Schematic representation of treatment of residual tumor after iRFA in C57/BL6 mice. **B** Bioluminescence images of mice with residual tumors after iRFA after different treatments on days 0, 10, and 20 (n = 3). **C** Bioluminescence signals of mice in each group on day 0, 10, and 20 (n = 3). **D** Tumor volume of residual tumors after iRFA in different groups(n = 6). **E** The weight of residual tumors on day 21 after different treatments (n = 6). **F** Survival analysis of experimental mice in different groups (n = 6). **G** Representative Flow cytometry plots and proportions of CD8^+^ T cells on day 7 (n = 6). **H** Representative flow cytometry plots and proportions of Granzyme B^+^ cells on day 7 (n = 6). ns, not significant *p < 0.05, **p < 0.01, ***p < 0.001

The efficacy of the combination of BT-NPs@PLEL and anti-PD-L1 treatment was further investigated. The experimental design follows the illustrated therapeutic schedule (Fig. 6A). Significantly stronger inhibition of tumor progression was observed in the combined group compared with that of the BT-NPs@PLEL and αPD-L1-treated group, as indicated by bioluminescence intensity measurements (Fig. 6B, C). Consistent with these results was indicated by the tumor growth curve (Fig. 6D). After a 21 day treatment, the combination therapy demonstrated superior effectiveness in treating residual tumors after iRFA. Remarkably, 1 out of 6 tumors was completely eradicated. In contrast, treatments with either BT-NPs@PLEL or anti-PD-L1 alone showed comparatively moderate effectiveness, with no instances of complete tumor eradication (Fig. 6E, Figure S16). This outcome was further corroborated by the survival analysis, which showed that mice subjected to the combination therapy experienced extended survival durations in the context of iRFA (Fig. 6F). Flow cytometry analysis of residual tumors was conducted on day 7 after different treatments. The results revealed that the group treated with the combination therapy had a significantly higher proportion of CD8^+^ T cells (Fig. 6G). Furthermore, these CD8^+^ T cells exhibited a higher expression of Granzyme B than those treated with BT-NPs@PLEL or anti-PD-L1 alone (Fig. 6H). Consistently, the residual tumors from the combination treatment group exhibited higher levels of pro-inflammatory cytokines, including IFN-γ and TNF-α (Figure S17). These findings suggest that BT-NPs@PLEL treatment and anti-PD-L1 therapy complement each other, leading to a combination therapy that enhances anti-tumor immunity.

### Combining BT-NPs@PLEL with anti-PD-L1 treatment enhanced effectiveness in inhibiting distant tumors

**Fig. 7:**
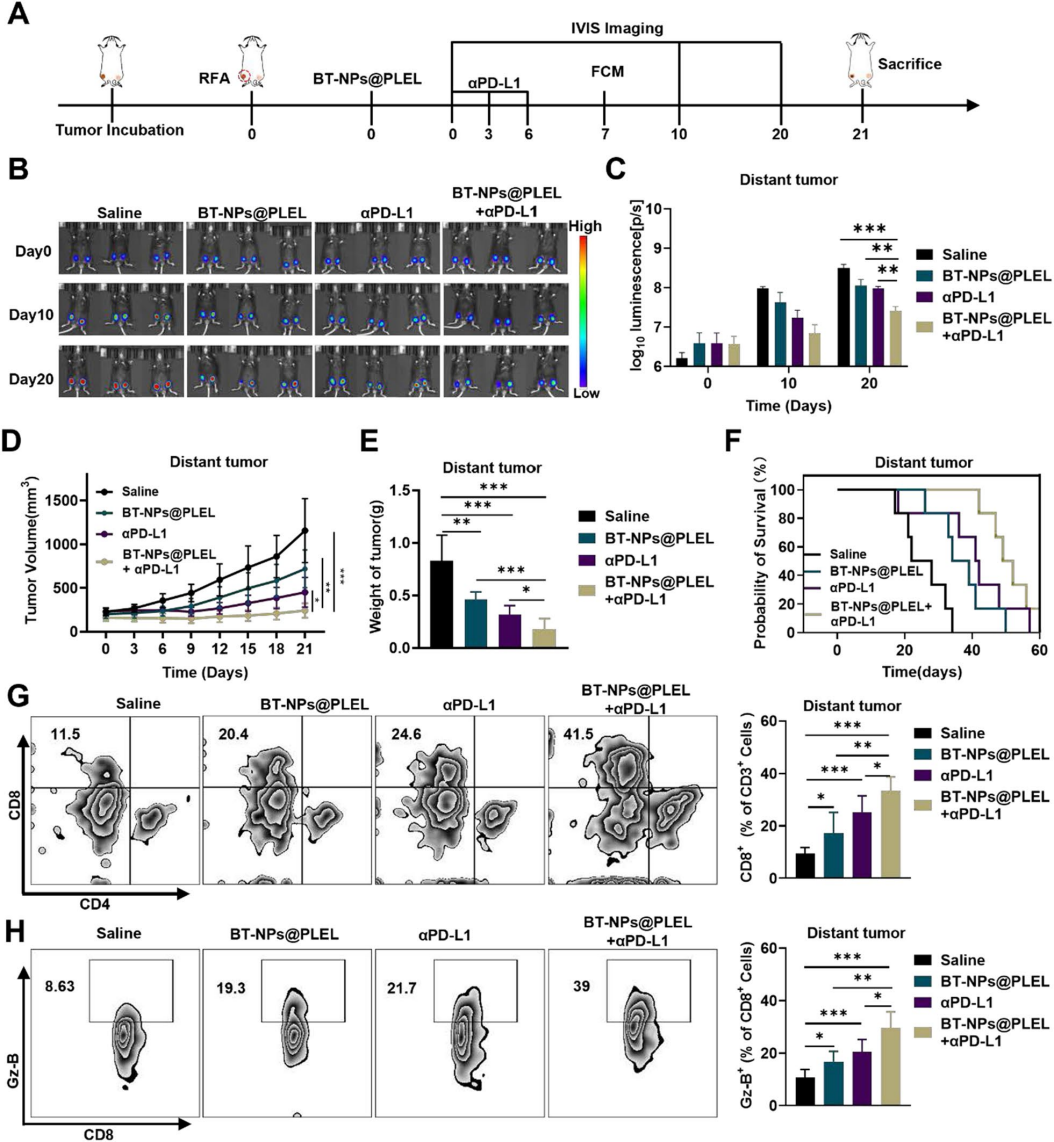
Combining BT-NPs@PLEL with anti-PD-L1 treatment for distant tumor inhibition. **A** Schematic representation of the treatment of residual tumors after iRFA and distant tumors in C57/BL6 mice. **B** Bioluminescence images of mice with residual tumors after iRFA and distant tumors after different treatments on days 0, 10, and 20 (n = 3). **C** Bioluminescence signals of mice in each group on day 0, 10, and 20. **D** Tumor volume of distant tumors in different groups (n = 6). **E** The weight of distant tumors post-iRFA on day 21 after different treatments (n = 6). **F** Survival analysis of experimental mice in different groups (n = 6). **G** Representative flow cytometry plots and proportions of CD8^+^ T cells in distant tumors on day 7 (n = 6). **H** Representative flow cytometry plots and proportions of Granzyme B^+^ cells in distant tumors on day 7 (n = 6). ns, not significant, *p < 0.05, **p < 0.01, ***p < 0.001

Occasionally, residual tumors after iRFA may coexist with distant metastasis or concurrent tumors [33]. Biomaterials-assisted local treatments have the potential to stimulate systemic tumor-specific immunological responses. These responses can be further enhanced when combined with Immune Checkpoint Blockade (ICB) therapy, which has the capability to fight metastatic cancer cells [34]. Consequently, BT-NPs@PLEL holds promise for augmenting the anti-tumor effectiveness of αPD-L1 therapy and suppressing distant tumors. Our study employed a bilateral tumor model to assess the systemic anti-tumor response, as depicted in the therapeutic schedule (Fig. 7A). As indicated by the bioluminescence imaging, the combination therapy notably suppressed the growth of distant tumors, while the efficacy of BT-NPs@PLEL and anti-PD-L1 treatments was comparatively moderate (Fig. 7B, C). Consistent with these results was indicated by the tumor growth curve (Fig. 7D). Following a 21 day treatment period, the combination therapy showed superior effectiveness in treating the distant tumor, as evidenced by a reduction in both tumor volume and weight (Fig. 7E, Figure S18).This result was further validated by the survival analysis, demonstrating that mice treated with the combination therapy had prolonged survival periods in scenarios involving iRFA with the presence of a distant tumor (Fig. 7F). To investigate whether the BT-NPs@PLEL and anti-PD-L1 combination suppressed the distant tumors by triggering a systemic anti-tumor response, we first evaluated the immune response in the spleens. Spleens from mice in the iRFA models were gathered on day7 following various treatments for flow cytometry analysis. We observed that the proportions and cytolytic function of CD8^+^ T cells were significantly increased in the combination treatment group (Figure S19), suggesting an activated adaptive systemic immune response. At the same time, the flow cytometry analysis of distant tumors showed that the proportions and cytolytic function of CD8^+^ T cells were significantly increased in mice receiving the combination treatment, while a weaker trend was observed in the BT-NPs@PLEL and anti-PD-L1 group, respectively (Fig. 7 G, H). Consistently, higher levels of cytokines (TNF-α, IFN-γ) were detected in the combination treatment group (Figure S20). These results indicate that the synergistic use of BT-NPs@PLEL and αPD-L1 can stimulate a heightened systemic anti-tumor immune response in residual tumor after iRFA.

## Discussion

In this study, the acceleration of residual tumor growth following iRFA was demonstrated in a murine model. The residual tumors exhibited a suppressive TIME, diminished IFN-1 signaling, and decreased DCs and CTLs. Concurrently, there was an increase in *Sumo2*  expression and activation of SUMOylation in these residual tumors following iRFA. Upon further investigation, we found that that SUMOylation, mediated by *Sumo2*, obstructed IFN-1 signaling in HCC cells following iRFA. This obstruction, in turn, hindered the anti-tumor immunity mediated by DCs and CTLs. Both the knockdown of *Sumo2 *and the pharmacological inhibition of SUMOylation resulted in the activation of IFN-1 signaling in HCC cells post-iRFA and subsequently promoted DC maturation in a co-culture system. However, this effect was reversed upon administration of an IFN-1 antibody. Given that DCs play a pivotal role in antigen presentation and the activation of CTLs [11], we posited that targeting SUMOylation could activate anti-tumor immunity and effectively treat residual tumors after iRFA. Thus, a small molecule SUMOylation inhibitor TAK-981-based nanocomposite hydrogel was devised to effectively target SUMO2-mediated SUMOylation within the residual tumors, named BT-NPs@PLEL. The in vivo results suggest that BT-NPs@PLEL shows superior efficacy in promoting the maturation of DCs and the infiltration and activation of CTLs, which result in enhanced suppression of residual tumors. Significantly, extensive PD-L1 upregulation was observed after BT-NPs@PLEL treatment in the residual tumors, setting the stage for the combination strategy with anti-PD-L1 treatment. Combining BT-NPs@PLEL with anti-PD-L1 treatment demonstrated a potent synergistic effect in activating the antitumor immune response, eliminating residual tumors after iRFA, and suppressing distant tumors. Collectively, this study identifies an innovative therapeutic target and develops a new strategy to treat RFA residual tumors, offering potential for eliminating remnant tumors and preventing HCC recurrence post-iRFA.

The overexpression of *Sumo2* and activating of SUMOylation were first reported in residual tumors following iRFA and were revealed to play a critical role in shaping suppressive TIME in this study. A similar pattern of *Sumo2* expression could also be found in the GEO database associated with iRFA (GSE138224) [5], signifying that this phenomenon is consistent rather than a random event. Indeed, SUMO mediated SUMOylation activation has long been linked to heat stress [35]. Studies have demonstrated that SUMO-2/3-mediated SUMOylation is upregulated after heat shock, a mechanism essential for cellular survival during stressful conditions. Our findings and the GEO database data consistently demonstrate a significant increase solely in *Sumo2* expression within residual tumors after iRFA, with no differential expression observed in other genes associated with SUMOylation signaling. The precise underlying mechanism for this specificity remains elusive. Research has proposed a potential connection involving Heat Shock Protein 27 (HSP27), which has been documented to augment the number of cellular proteins modified by SUMO2/3. It has also been linked to the SUMO2/3 modification of Heat Shock Factor 1 (HSF1), thereby modulating the activity of this transcription factor [36]. Further investigation is warranted to ascertain whether HSP27 regulates the translational expression of SUMO2.

In our investigation, we observed a notable inhibition of the IFN-1 signaling pathway within RFA residual tumors. Upon knocking down *Sumo2* or inhibiting SUMOylation, we observed a significant upregulation of IFN-1 signaling in the HCC cells post-iRFA. This finding aligns with existing research that suggests SUMO proteins can modify several targets critical for IFN-1 signaling, including, cGAS, IRF3, IRF7, and the enhancer element of IFN-β [14, 37, 38]. These studies collectively highlight that SUMOylation of these targets negatively regulates IFN-1 production. Our findings indicated a poor infiltration of mature DCs and CD8^+^ T cells, suggesting inhibited antigen presentation in residual tumors after iRFA. Given the crucial role of the IFN-1 pathway in DC functions and antigen presentation for the host’s antitumor immune response, targeting SUMOylation emerges as a promising approach to activate DCs. Indeed, our results suggest that inhibiting SUMOylation effectively activated DCs in both in vivo and in vitro models. In this study, we primarily explored the SUMOylation/IFN-1 mechanism in HCC cells under heat stress, as the sequencing results primarily reflected the overall state of the iRFA tumor. Additionally, previous research by Lu C et al. has highlighted the pivotal role of IFN-1 signaling within tumor cells for DC-mediated adaptive antitumor immunity, while Wang Z et al. reported that activating IFN-1 signaling in tumor cells contributes to the activation of dendritic cells and subsequent T cells [39, 40]. These cumulative findings emphasize the significance of IFN-1 signaling within tumor cells in orchestrating the anti-tumor immune response and underscore the potential of targeting SUMOylation as a therapeutic strategy for treating residual tumors after iRFA.

TAK-981, a SUMOylation inhibitor, was explored as a potential treatment for residual HCC after iRFA. TAK-981 has demonstrated promising preclinical efficacy in activating anti-tumor immunity [20, 41–44], prompting its evaluation in several phase 1/2 clinical trials for various kinds of tumors (#NCT03648372, #NCT04074330, #NCT04776018, and #NCT04381650), but not for HCC. However, the broad application of TAK-981 is challenged by important factors that need to be considered, particularly the potential toxicity risks associated with TAK-981 due to the widespread presence of SUMO modification. Systemic and local injections of TAK-981 can induce undesirable inflammation in normal tissues, leading to adverse events such as diarrhea and ulceration [20]. Additionally, its poor solubility and low bioavailability further limit its clinical utility. Another challenge is the inability of systemic or local injections to maintain sustained, high local drug concentrations, which is essential for the effective eradication of residual tumors in iRFA cases. To overcome these limitations, we have developed a novel injectable drug delivery system that combines BSA-based nanoparticles with a thermosensitive PLEL hydrogel. This innovative method ensures the encapsulation and localized, sustained release of TAK-981, enhancing its effectiveness against RFA residual tumors. The system’s key advantage lies in its ability to undergo a spontaneous sol–gel transition upon heating, minimizing systemic drug exposure. Although hydrogels are inherently hydrophilic, posing challenges in loading hydrophobic agents due to their high water content [45], our strategy utilizes BSA-based nanoparticles to overcome these challenges. These nanoparticles effectively encapsulate TAK-981, elevating drug loading capacity, enhancing solubility, and circumventing the obstacles associated with integrating hydrophobic agents into hydrogel systems.

Immunotherapy, particularly anti-PD-1/PD-L1 therapy, has revolutionized the landscape of cancer treatment over the past decade. However, it’s worth noting that the response rate for advanced HCC stands at a modest 18.3% [46]. The efficacy of anti-PD-1/PD-L1 therapy relies significantly on the expression of PD-1/PD-L1 in TIME [44, 47–50]. Our research, corroborated by other studies, has substantiated that administering TAK-981 treatment results in an augmented expression of PD-L1 at the cellular level within tumor tissues [21]. The heightened PD-L1 expression potentially influences the treatment efficacy of TAK-981 nanocomposite hydrogel, aligning with the prerequisites for anti-PD-1/PD-L1 treatment. Our data suggest that combining BT-NPs@PLEL with anti-PD-L1 treatment significantly enhances the efficacy in treating residual tumors after iRFA and distant tumors, indicating a potential for an extended application of BT-NPs@PLEL.

## Conclusions

In summary, this study represents a significant breakthrough by unveiling the role of SUMO2-mediated SUMOylation, induced by iRFA, in hampering IFN-1 production and impeding antigen presentation in residual tumors. This discovery introduces a novel therapeutic target, suggesting that targeting SUMOylation might enhance immune surveillance and offer a potential avenue to prevent HCC recurrence post-RFA treatment. Additionally, the development of an injectable drug delivery hydrogel (BT-NPs@PLEL) shows promising prospects to amplify the efficacy and broaden the clinical applications of the SUMOylation inhibitor. The exploration of the combined strategy of BT-NPs@PLEL and anti-PD-L1 presents opportunities to expand the application of RFA and enhance the response rate of immunotherapy.

## Reagents and methods

### Reagent and equipment

TAK-981(S8829) was purchased from Selleck Co Ltd. SUMO-2/3 (#4974) and STAT1 (14994 T) antibodies were purchased from Cell Signaling Technology (USA), Rabbit Anti-Sumo2 antibody (bs-15494R) was purchased from Bioss Antibodies, and Phospho-STAT1 (Tyr727) antibody (ab109461) was purchased from Abcam plc (USA). PDLLA-PEG-PDLLA (R-PL1054) was purchased from Xi’an Ruixi Biological Technology Co Ltd. InVivoMab anti-mouse PD-L1 (B7-H1) (BE0101) was purchased from BioXcell Technologies, Inc (USA). VIVA RFA system (VRS01, STARmed) with a straight RFA probe was used for RFA treatment.

### Cell lines and animals

The Hepa1-6, Hep3B, HepG2 cells were originally obtained from the American Type Culture Collection and were grown in Dulbecco’s Modified Eagle Medium (DMEM) supplemented with 1% Penicillin–Streptomycin and 10% fetal bovine serum (FBS). The cells were maintained under standard conditions at 37 °C with 5% CO_2_. Female C57/BL6 mice, aged 5–6 weeks, were procured from the Medical Laboratory Animal Center of Guangdong Province. All experiments involving mice were carried out in accordance with the guidelines set forth by the Institutional Animal Care and Use Committee of The Fifth affiliated hospital of Sun Yat-Sen University.

### *Sumo2* gene knockout in Hepa1-6

The *Sumo2* gene was knocked down by short hairpin RNA (shRNA) in Hepa1-6 cells. The shRNA and vector design and construction were performed by IGE Biotechnology LTD. In brief, shRNAs were designed against the m*Sumo2* gene (Mus musculus, NC_000077.7) using CHOPCHOP. Lentivirus packaging was carried out, and Hepa1-6 cells were transduced according to the abm protocol. Puromycin selection was completed two days after transduction.

### Total RNA extraction and real-time PCR

Total RNA was collected and isolated from tissues and cells with an RNA isolater Total RNA Extraction Reagent kit (Vazyme Biotech Co., Ltd, Nanjing, China). Reverse transcription was performed using the HiScript III 1st Strand cDNA Synthesis Kit (Vazyme Biotech Co., Ltd, Nanjing, China). The PCR was performed by a ChamQ Blue Universal SYBR qPCR Master Mix (Vazyme Biotech Co., Ltd, Nanjing, China), according to the manufacturer’s instructions. The primers were designed according to the National Center for Biotechnology Information (NCBI) database using the Primer Premier 5.0 software (Palo Alto, CA, USA), and all the primer sequences are listed in Table 1. The mRNA expression was measured using a real-time PCR machine, ABI 7500 (Applied Biosystems, USA). Fold changes expression of each gene was calculated by the comparative threshold cycle (Ct) method using the formula 2^− (ΔΔCt)^.

**Table 1:** Selection of primer sequences of genes for RT-qPCR Analysis

Gene	The primer sequence of gene for RT-qPCR analysis	The version of nucleotide sequence
	Primer sequence(5'-3')	
*Sumo2*	*Forward*: CCCggTgCCTCTTTTgTgAA *Reverse*: gACTCCTTCCTTgggTTTCTCg	NM_133354.2
SUMO2	*Forward*: gACgAAAAgCCCAAggAAggAg *Reverse*: CCATTTCCAACTgTCgTTCACA	NM_001005849.2
*Ifnb1*	*Forward*:gCAAgAggAAAgATTgACgTgg *Reverse*: AggCgTAgCTgTTgTACTTCAT	NM_010510.2
*Isg15*	*Forward*: CAgCAATggCCTgggACCTAAA *Reverse*: gCACACCAATCTTCTgggCAAT	NM_015783.3
*Ifit3*	*Forward*: TCAgCCCACACCCAgCTTTT *Reverse*: CTTCCAgAgATTCCCggTTgAC	NM_010501.2
*Mx1*	*Forward*: gCAgAAgTACggTgCAgACATA *Reverse*: ACggTTTCCTgTgCTTgTATCA	NM_010846.1
*Cxcl10*	*Forward*: CCgTCATTTTCTgCCTCATCCT *Reverse*: TTCCCTATggCCCTCATTCTCA	NM_021274.2
*Il6*	*Forward*: ACAgAggATACCACTCCCAACA *Reverse*: gCCATTgCACAACTCTTTTCTCA	NM_001314054.1
*Gapdh*	*Forward*: ATgACATCAAgAAggTggTg *Reverse*: CATACCAggAAATgAgCTTg	NM_001289726.2
GAPDH	*Forword*: ACAACTTTggTATCgTggAAgg *Reverse*: gCCATCACgCCACAgTTTC	NM_001256799.3

### RNA-sequencing (RNA-seq) and bioinformatics analysis

RNA-seq libraries were performed by Majorbio Corporation (Shanghai, China), and the data were expressed as the means displayed in the center of the heatmaps. The fold-change was calculated and converted to log2. All mRNA sequencing data were downloaded from the TCGA data portal.

### Western blot analysis

An equivalent of 30–50 μg total cellular protein was separated by a 10–15% gradient in SDS-PAGE (Bio-Rad Laboratories). The proteins were transferred to nitrocellulose membrane (Pall Corporation, Ann Arbor, MI, USA), and the membranes were blocked with 5% milk powder in TBST for 60 min. Then, the blots were probed in 0.1% casein/TBS-T with Primary antibody overnight at 4° C. Subsequently, the blots were incubated with infrared-labeled secondary Abs at 1:5000 at room temperature for 1 h. The immunoreactive bands were visualized using an Invitrogen iBright^™^ CL750 Gel Imaging System (Thermo Fisher Scientific, USA).

### Immunohistochemistry (IHC) staining

Immunohistochemistry (IHC) staining of paraffin-embedded tumor tissue sections was performed using the anti-SUMO2 (bs-15494R, Bioss) antibodies, according to the manufacturer’s instructions. For statistical analysis, the DAB staining score was calculated by Image J with the IHC Profiler plugin [51]. The score of the zone is assigned as 4 for the high positive zone, 3 for the positive zone, 2 for the low positive zone and 1 for the negative zone. The score calculation following the algebraic formula:

1 $$\begin{array}{c}score=\frac{\left(\text{Number of pixels in a zone}\right)\left(\text{Score of the zone}\right)}{Total \,number \,of \,pixels \,in \,the \,image}\end{array}$$

### Transwell experiment on DCs stimulation in vitro

BMDCs were obtained from the marrow of the tibia and femur of C57BL/6 mice by flushing with RPMI 1640 medium. The red blood cells were then lysed, and the remaining cells were cultured in a medium containing 20 ng/mL GM-CSF and 10 ng/mL IL4 for 7 days. The expression of CD11c was assessed before the experiment. Heated Hepa1-6 cells were cultured in the upper compartment of the transwell system, while the BMDCs were co-seeded in the lower compartment. After various treatments, BMDCs stained with APC-anti-CD45, PE/Dazzle-anti-CD11c, Percp/Cy5.5anti-CD86 PE, and PE/Cy7-anti-CD80 were analyzed by flow cytometry.

### Construction and of TAK-981@BSA nanoparticles

With minimal alteration, the TAK-981 loaded BSA nanoparticles were created using a self-assembly technique in accordance with earlier research [52]. In brief, dimethyl sulfoxide (DMSO) solution of 100 μL TAK-981(30 mg/mL) was added into the solution of BSA (9 mg/mL, 10 mL, pH 8.0, adjusted with 0.1 M NaOH) and stirred vigorously for 2 h. The resulting nanoparticles were gathered through gradient centrifugation. To remove the excess unbound drug, we performed centrifugal filtration using an Amicon filter (MWCO = 10 kDa) and washed the sample three times with PBS until no drug was detected in the filtrate. The morphology of TAK-981@BSA NPs was characterized with a transmission electron microscope (FEI Tecnai F20). The diameters of BT-NPs@PLEL were detected by Zeta sizer Nano-ZS (Malvern Instruments, UK).

### Fabrication of BT-NPs@PLEL composite hydrogel

The prepared TAK-981@BSA nanoparticles were dispersed into a 100 μL solution and were incorporated with a 400 μL PLEL solution to construct the final BT-NPs@PLEL composites (PLEL, 25 wt%). Additionally, the initial TAK-981 loading was determined by measuring the concentration in the discarded supernatant and comparing it to the concentration prior to the formation of NPs.

### The accumulative releaseof drugs from BT-NPs@PLEL

The accumulative drug release behavior was measured using a transwell system in PBS containing 20% fetal bovine serum at 37° C to mimic the physiological environment. The BT-NPs@PLEL (25 wt%) encapsulated with TAK-981 (0.5 mg) was loaded into the upper chamber, and the released TAK-981 was collected from the bottom of chamber at various time points and quantified using a PerkinElmer Lambda 750 UV–vis–NIR spectrophotometer.

### Phase diagram of PLEL and BT-NPs@PLEL

The precursor solution of PLEL and BT-NPs@PLEL with concentrations of 25 wt% was prepared, then 1 mL of each solution was added into a vial and equilibrated in the water bath for 10 min at each temperature. The gel state was determined by inverting the vial and observing whether the solution flowed within 30 s.

### The in situ gelation and degradation behavior of PLEL

For an in situ gelation experiment, the precursor solution of BT-NPs@PLEL with a concentration of 25 wt% was injected subcutaneously. Then, the skins of mice were sacrificed and imaged. For an in vivo degradation experiment, 100 μL BT-NPs@PLEL (mixed with blue dye) (25 wt %) was injected into the flank of the mouse. Then the skins of mice were sacrificed and imaged on day 1, 7, and 14 for records of in vivo biodegradability.

### Flow cytometry analysis

Single cells were obtained by homogenizing the tumors and spleen on day 7 after treatment. Then, the cells were blocked with anti-CD16/32 (Elabscience, E-AB-F0997A) antibodies to avoid nonspecific adsorption and then stained with the following antibodies according to the specification: APC-anti-CD45 (BioLegend, 103112), PE/Dazzle-anti-CD11c (BioLegend, 117347), PE/Cy7-anti-CD80 (BioLegend, 104733), Percp/Cy5.5-anti-CD86 (BioLegend, 105027), PE-anti-CD86 (BioLegend, 105008), anti-CD45 (BioLegend, 103132), APC/Cy7-anti-CD3 (BioLegend, 100222), BV605-anti-CD4 (BioLegend, 100548), BV510-anti-CD8 (BioLegend, 100751), APC-anti- Granzyme B (BioLegend, 372204). 5.0 × 10^3^ events in the CD45 flow chart in each sample were collected using a CytoFLEX LX Flow Cytometer (Beckman Coulter, USA) and analyzed by FlowJo 10.8.1. The gating strategy is given in Figure S21, Supplementary material.

### In Vivo experiment

To build the HCC model, 1.0 × 10^^6^ Hepa1-6 cells were subcutaneously inoculated on the right flank or bilateral of each C57BL/6 mouse. 14 days later (Tumor volume about 300 mm^3^), the mice were randomly divided into different groups to perform in vivo experiments. To study the drug retention in tumors, equal amounts of free Sulfo-Cyanine5.5 dye and Cy5.5@Gel were injected subcutaneously, and the fluorescence signal was monitored by a fluorescence imaging system (PerkinElmer, Lumina). For RFA treatment, the radiofrequency needle was pierced into a non-central location of the tumor to achieve partial necrosis of the target tumors (Figure S1A), and the power of radiofrequency was 7 Walt, and the tumor was heated to 60° C for 0.5 min. Then, the equivalent TAK-981 (150 μg per mouse) and gel (85 μL, 25 wt% per mouse) were injected into the tumor in the corresponding group. The tumor growth curves were monitored by calculating the volume using the following formula:

2 $$V=\frac{({w}^{2}\times L)}{2}$$

All animal experiments are conducted after administering inhalation anesthesia with isoflurane.

### In Vivo biosafety evaluation

To evaluate the biosafety of BT-NPs@PLEL in vivo, four groups of healthy mice were injected subcutaneously with BT-NPs@PLEL (TAK-981, 7.5 mg/kg) and sacrificed on day 1, day 10, and day 30, respectively, while the control group received PBS injection alone. Blood items including white blood cells (WBC), red blood cells (RBC), platelets (PLT), Total protein (TP), serum albumin (ALB), urea nitrogen (Urea), creatinine (Crea), aspartate aminotransferase (AST), alanine aminotransferase (ALT) and albumin (ALB) were tested.

### Cytokine detection

The tumors were harvested and homogenized thoroughly in RIPA lysis buffer at 4° C on day 7 and centrifuged (12,000 rpm) to remove the sediment 3 times. The concentration of cytokines was detected by IFN-γ (MIKX, SZ1095), and TNF-α (MIKX, SZ1098) mouse ELISA assay kit according to the manufacturer’s instructions.

### Statistical analysis

Statistical analysis was performed via GraphPad Prism 9.0. All data are presented as the mean ± standard error of the mean (s.e.m.). The Two-tailed Student’s t-test was used for two group comparisons, One-way ANOVA (for multiple groups) and two-way ANOVA (for multiple groups and factors) were used for multiple group comparisons, as appropriate, followed by Tukey’s post hoc multiple comparisons test. The threshold for statistical significance was ns, not significant, *p < 0.05, **p < 0.01, ***p < 0.001.

### Supplementary Information

Supplementary material 1.

## Data Availability

Data will be made available on request.

## Abbreviations

HCC: Hepatocellular carcinoma
RFA: Radiofrequency ablation
iRFA: Incomplete radiofrequency ablation
TIME: Tumor immunosuppressive microenvironment
SUMO2: Small ubiquitin-like modifier 2
PLEL: Poly (d, L-lactide)-poly (ethylene glycol)-poly (d, L-lactide) (PDLLA-PEG-PDLLA)
DCs: Dendritic cells
CTLs: Cytotoxic T-lymphocytes
IFN-1: Type I interferon
DMEM: Dulbecco’s modified eagle medium
FBS: Fetal bovine serum
IHC: Immunohistochemistry
NPs: Nanoparticles
WBC: White blood cells
RBC: Red blood cells
PLT: Platelets
TP: Total protein
ALB: Serum albumin
Urea: Urea nitrogen
Crea: Creatinine
AST: Aspartate aminotransferase
ALT: Alanine aminotransferase
ALB: Albumin
ISGs: Interferon-stimulated genes
BMDCs: Bone marrow-Derived dendritic cells
BSA: Bovine serum albumin
ICB: Immune checkpoint blockade
TNF-α: Tumor necrosis factor alpha
IFN-γ: Interferon gamma
