# Correction:Targeting SUMOylation with an injectable nanocomposite hydrogel to optimize radiofrequency ablation therapy for hepatocellular carcinoma

**DOI:** 10.1186/s12951-024-02690-3

**Published:** 2024-08-02

**Authors:** Junfeng Liu, Xi Li, Jiawen Chen, Jingpei Guo, Hui Guo, Xiaoting Zhang, Jinming Fan, Ke Zhang, Junjie Mao, Bin Zhou

**Affiliations:** 1 Center of Interventional Medicine, The Fifth Affiliated Hospital of Sun Yat-Sen University, Zhuhai, 519000 Guangdong Province China; 2 Institute of Interventional Radiology, Sun Yat-Sen University, Zhuhai, 519000 Guangdong Province China; 3 Center of Cerebrovascular Disease, The Fifth Affiliated Hospital of Sun Yat-Sen University, Zhuhai, 519000 Guangdong Province China; 4 Guangdong Provincial Engineering Research Center of Molecular Imaging, The Fifth Affiliated Hospital of Sun Yat-Sen University, Zhuhai, 519000 Guangdong Province China

**Correction: Journal of Nanobiotechnology (2024) 22:338** 10.1186/s12951-024-02579-1

In this article the wrong figure appeared as Fig. 6: author corrections in the title of the graph in panel E and in the image in panel H were not implemented due to a typesetting mistake. Figure 6 should have appeared as shown below.

Uncorrected figure
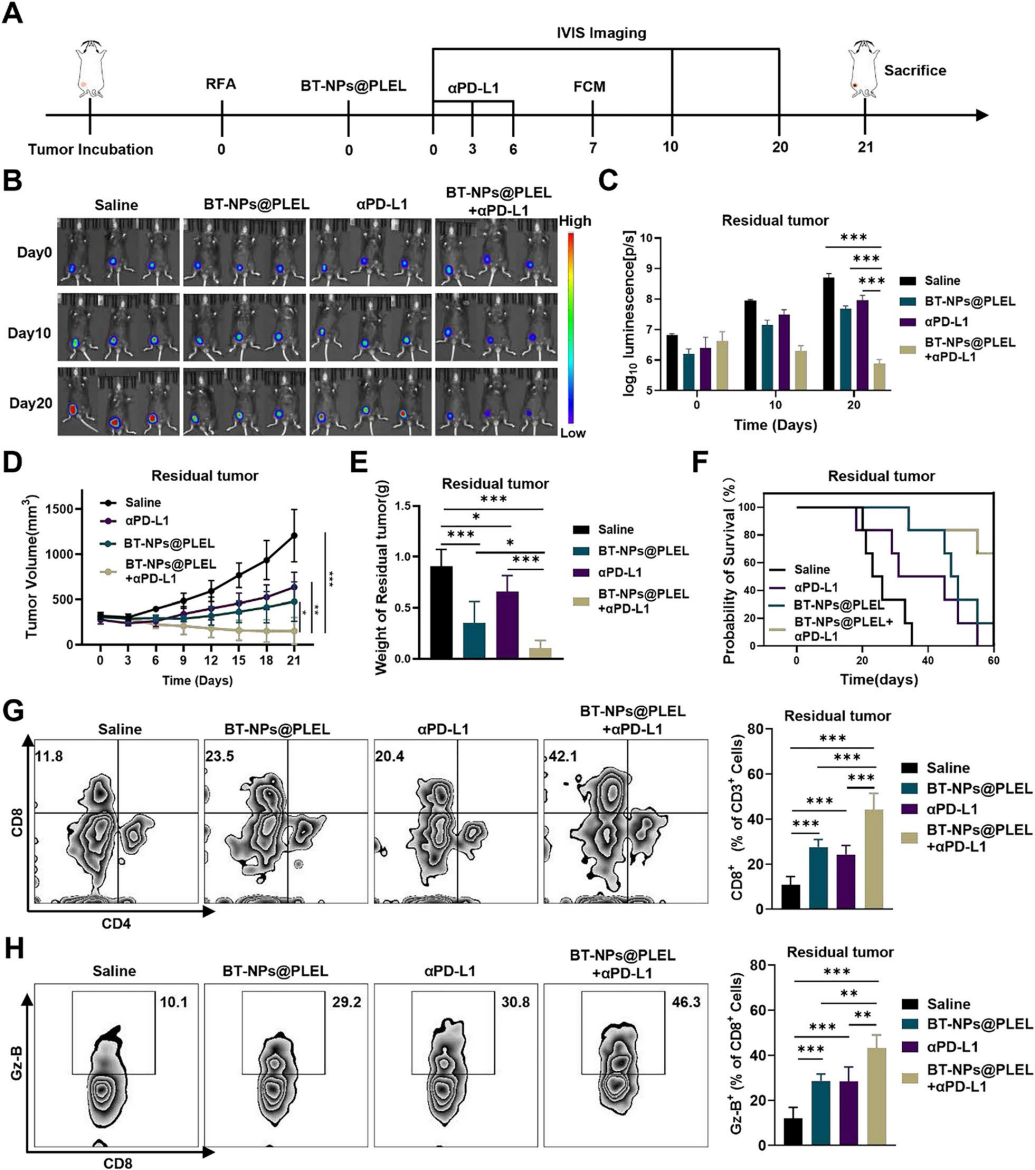


Corrected figure

**Fig. 6:**
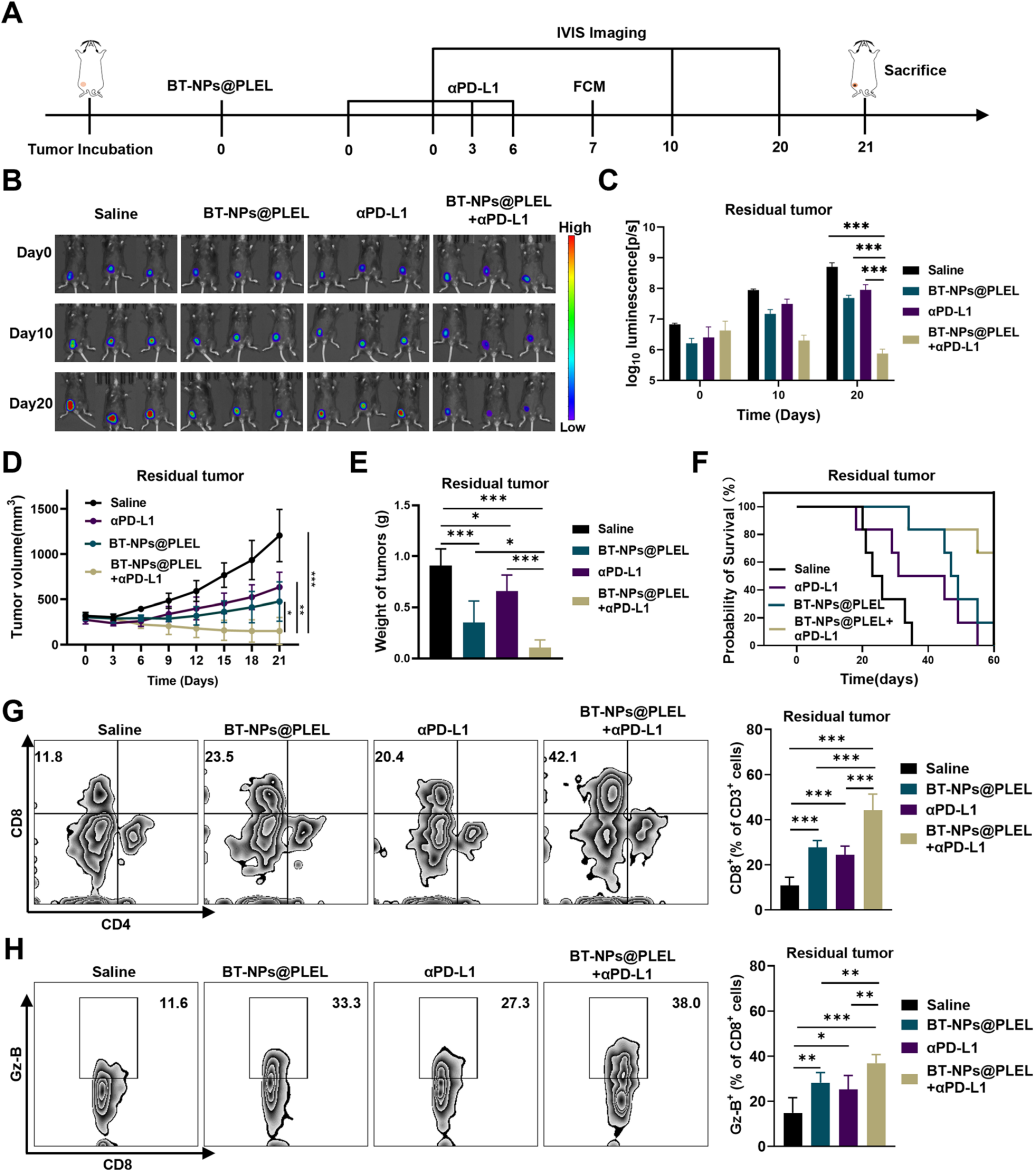
Combining BT-NPs@PLEL with anti-PD-L1 treatment for inhibition in residual tumors after iRFA. **A** Schematic representation of treatment of residual tumor after iRFA in C57/BL6 mice. **B** Bioluminescence images of mice with residual tumors after iRFA after different treatments on days 0, 10, and 20 (n = 3). **C** Bioluminescence signals of mice in each group on day 0, 10, and 20 (n = 3). **D** Tumor volume of residual tumors after iRFA in different groups(n = 6). **E** The weight of residual tumors on day 21 after different treatments (n = 6). **F** Survival analysis of experimental mice in different groups (n = 6). **G **Representative Flow cytometry plots and proportions of CD8+ T cells on day 7 (n = 6). **H** Representative flow cytometry plots and proportions of Granzyme B+ cells on day 7 (n = 6). ns, not significant *p < 0.05, **p < 0.01, ***p < 0.001

The original article has been corrected. The publisher apologises to the authors and readers for the inconvenience caused by this error.

