# Supplementary material for: Targeting SUMOylation with an injectable nanocomposite hydrogel to optimize radiofrequency ablation therapy for hepatocellular carcinoma
Source: J Nanobiotechnology. 2024 Jun 18;22:338. doi: 10.1186/s12951-024-02579-1 (PMC11184877; doi:10.1186/s12951-024-02579-1)
Supplement: Supplementary file 1 — Supplementary material 1. [file 12951_2024_2579_MOESM1_ESM.docx]

**Targeting SUMOylation with an Injectable Nanocomposite Hydrogel to Optimize Radiofrequency Ablation Therapy for Hepatocellular Carcinoma**

**Junfeng Liu^1, 2, 3, 4#^, Xi Li^1, 2, 4#^, Jiawen Chen^1, 2, 3, 4#^, Jingpei Guo^1, 2, 3, 4,^ , Hui Guo^1, 2, 4,^ , Xiaoting Zhang^1, 2, 3, 4^, Jinming Fan^1, 2, 3, 4^, Ke Zhang^1, 2, 4^, Junjie Mao^1, 2, 4^*, Xi Li^1, 2, 4*^, Bin Zhou^1, 2, 3, 4^***

^1^ Center of Interventional Medicine, The Fifth Affiliated Hospital of Sun Yat-sen University, Zhuhai, Guangdong Province, 519000, China

^2^ Institute of Interventional Radiology, Sun Yat-Sen University, Zhuhai, Guangdong Province, 519000, China

^3^ Center of Cerebrovascular Disease, The Fifth Affiliated Hospital of Sun Yat-sen University, Zhuhai, Guangdong Province, 519000, China

^4^ Guangdong Provincial Engineering Research Center of Molecular Imaging, The Fifth Affiliated Hospital of Sun Yat-sen University, Zhuhai, Guangdong Province, 519000, China

^#^J. F. Liu, X. Li, J. W. Chen contributed equally to this work.

* Corresponding authors:

Bin Zhou, M.D, Ph.D.

Junjie Mao, M.D., Ph.D.

Xi Li, M.D, Ph.D.

Center of Interventional Medicine, The Fifth Affiliated Hospital,

Sun Yat-sen University, Zhuhai, Guangdong Province, 519000, China

Email addresses for correspondence:

zhoub2@mail.sysu.edu.cn

lixi57@mail.sysu.edu.cn

[maojunj@mail.sysu.edu.cn](mailto:maojunj@mail.sysu.edu.cn)


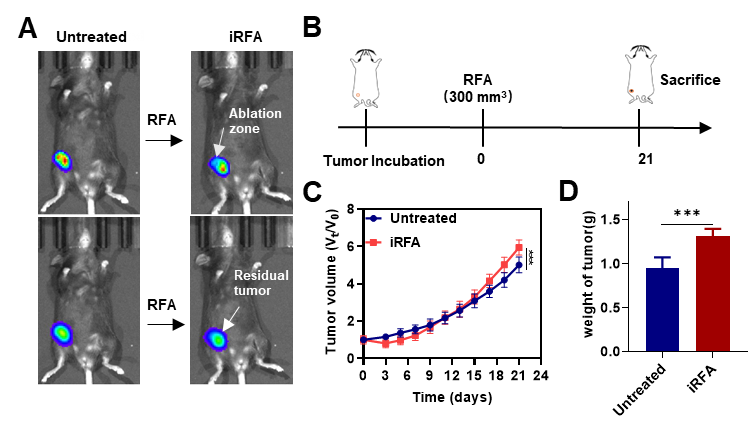


**Fig S1. iRFA causes rapid progression of residual tumor. A.** Schematic of the process of establishing an residual tumor model of HCC by *in vivo* bioluminescence imaging. **B.** Schematic representation of RFA treatment in HCC model in C57/BL6 mice. **C.** The growth curve of tumor volumes. **D.** Statistical analysis of the tumor weight on day 21. ****p* < 0.001.


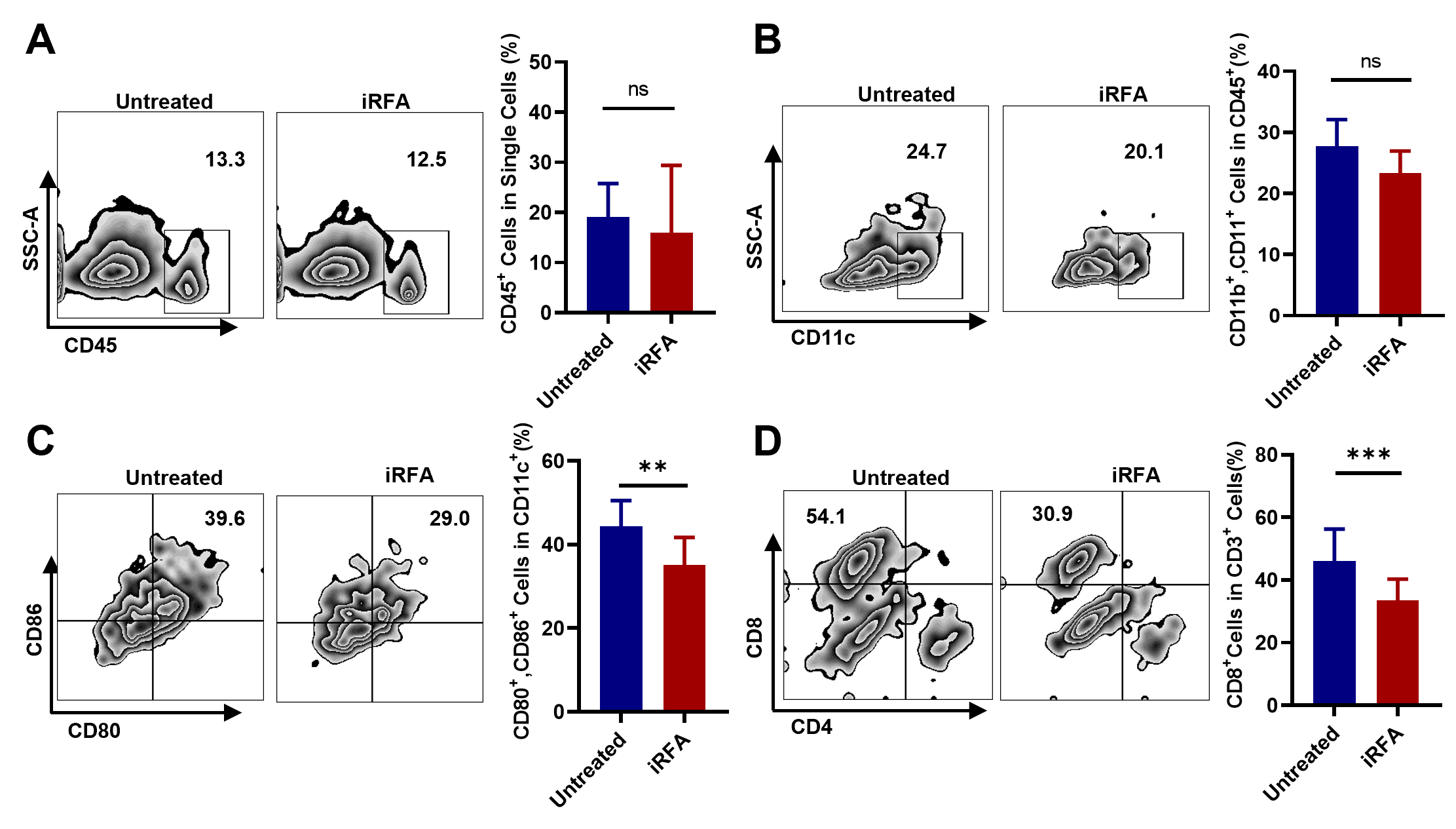


**Fig S2.** Formation of an immunosuppressive microenvironment in the residual tumors after iRFA. A. Representative flow cytometry plots and proportions of leukocytes on day 21 (n = 6). B. Representative flow cytometry plots and proportions of DCs on day 21 (n = 6). C. Representative flow cytometry plots and proportions of mature DCs on day 21 (n = 6). D. Representative flow cytometry plots and proportions of CD8^+^ T cells on day 21 (n = 6). ns, not significant, ***p* < 0.01, ****p* < 0.001.

**
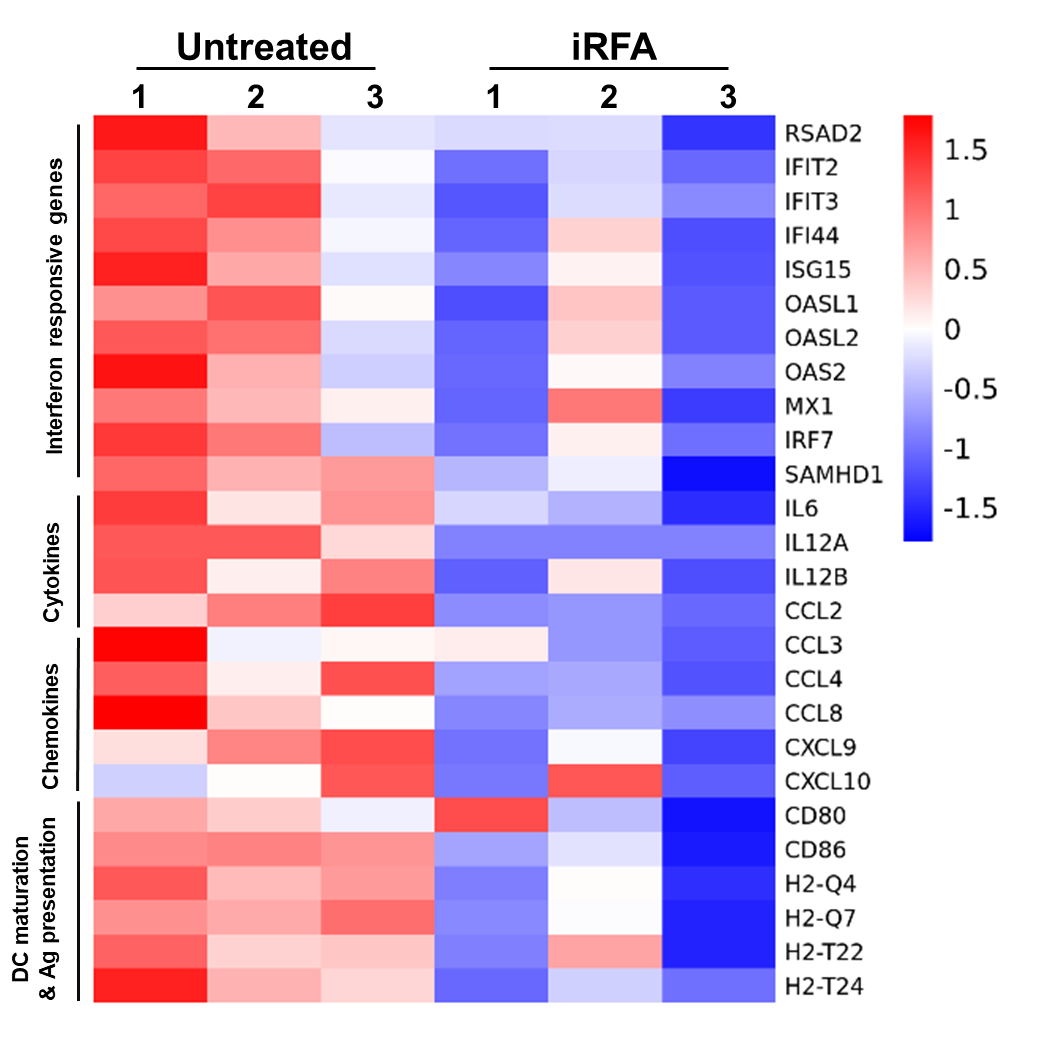
**

**Figure S3.** The heatmap of expressions of ISGs in the untreated and iRFA groups.

**
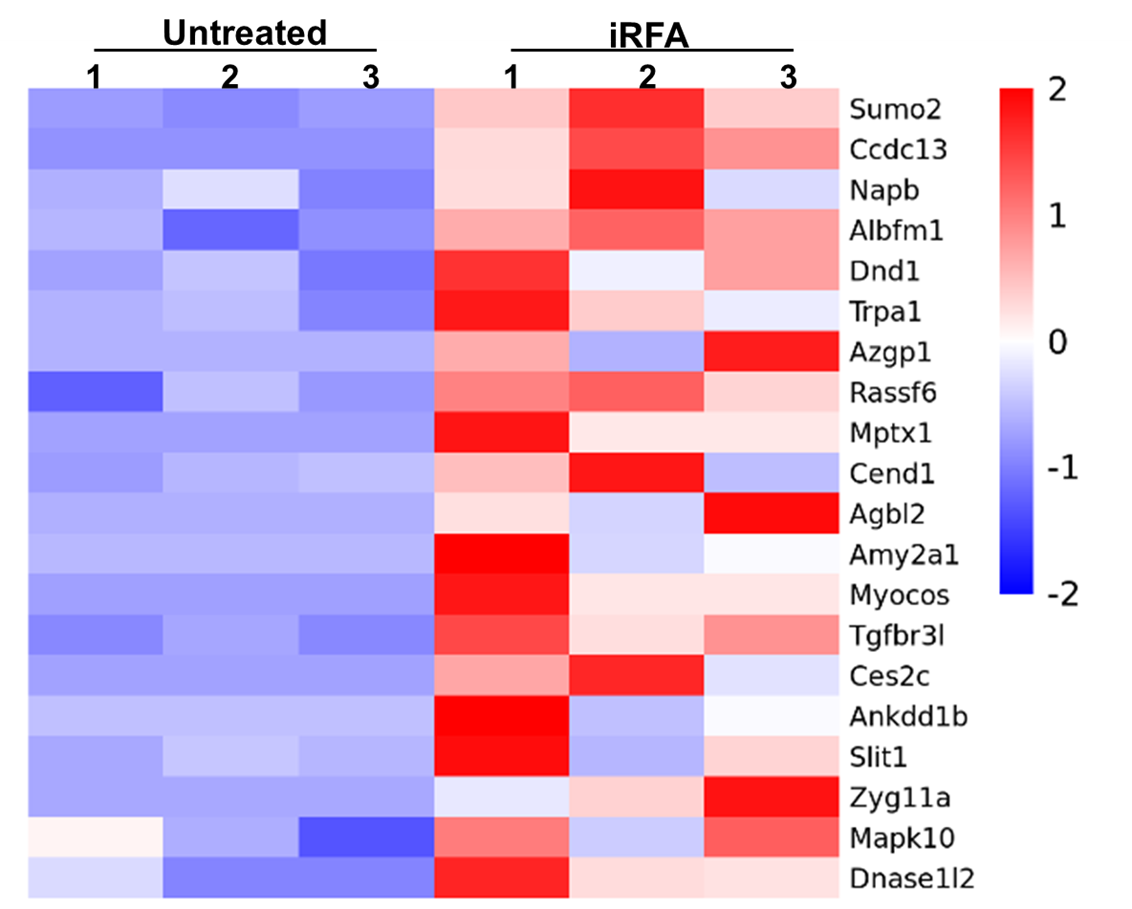
**

**Figure S4.** The heatmap of upregulated genes in the RNA-seq dataset.


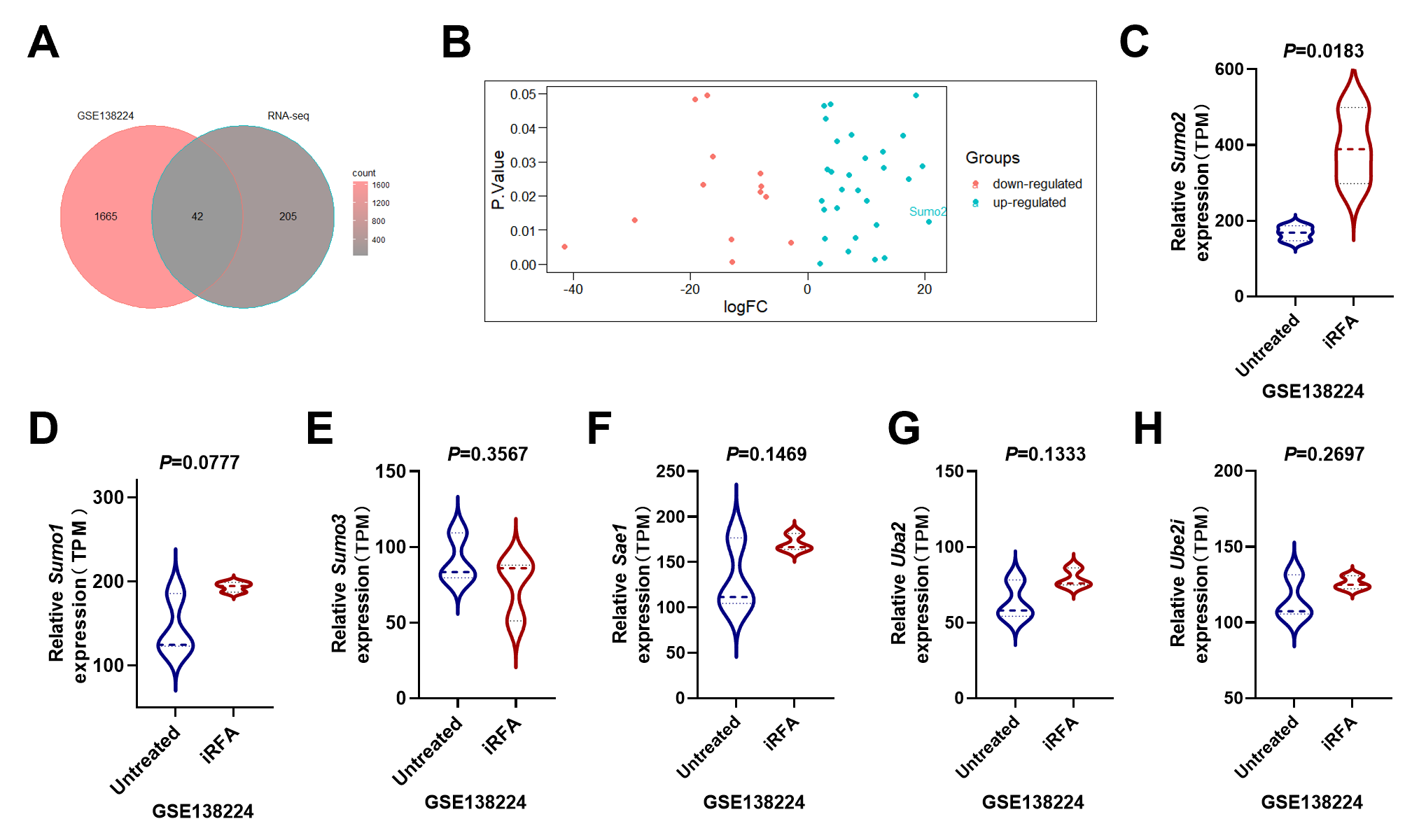


**Figure S5.** Upregulation of *Sumo2* in the residual tumor following iRFA treatment of colorectal cancer liver metastases (GEO sequencing database, GSE138224). **A.** Comparison of gene variant profiles between the GEO data cluster (GSE 138224) and the RNA-Seq data. **B.** Differences in intersecting genes between untreated and iRFA-treated groups. **C-H.** Differences in the expression of critical regulators in the SUMOylation pathway between the iRFA-treated and untreated groups (n=3).

**Figure S6.** Kaplan–Meier curves of overall survival of patients with HCC associated with *SUMO2* expression in the TCGA database.

**
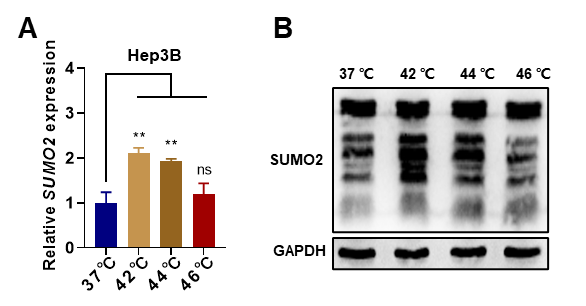
**

**Figure S7** The *SUMO2* expression and conjugated-SUMO2 increased in heated Hep3B cells. ns, not significant, ***p* < 0.01.

**Figure S8.** ELISA analysis of the IFN-β in the tumor tissue (n = 6 ). **p* < 0.05.


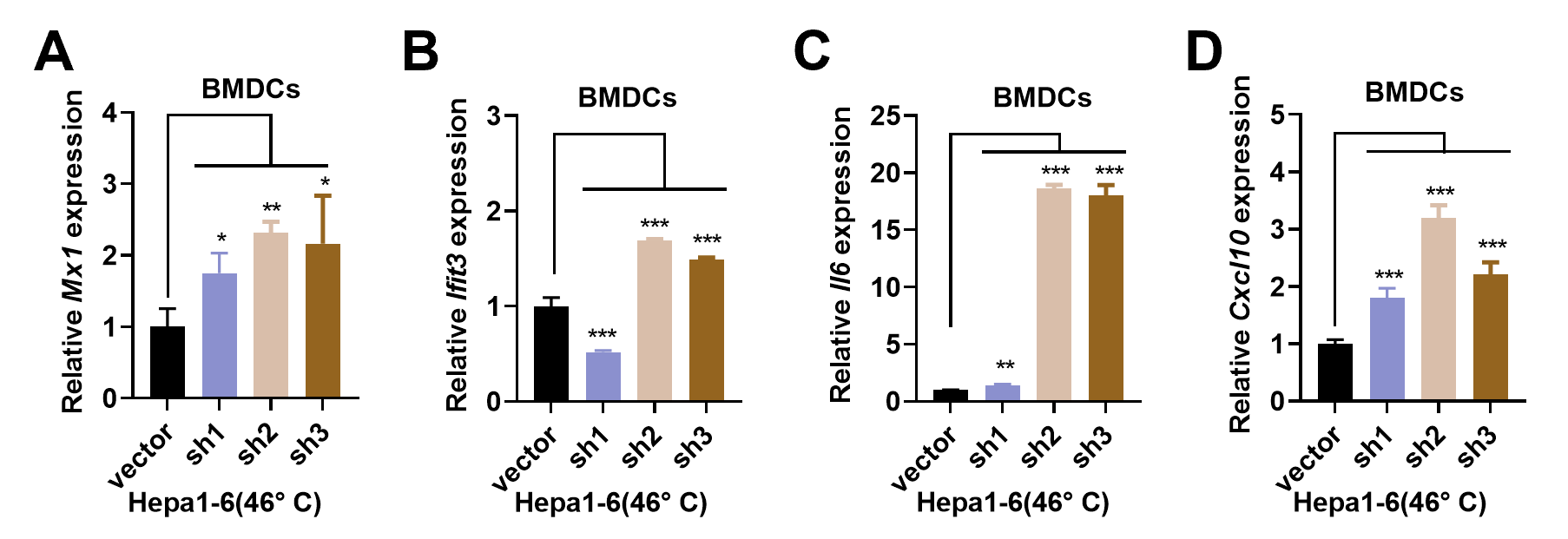


**Figure S9.** Relative expression of ISGs in BMDCs co-cultured with heated *Sumo2*-konckdown Hepa1-6 cells. ns, not significant, **p* < 0.05, ***p* < 0.01, ****p* < 0.001.


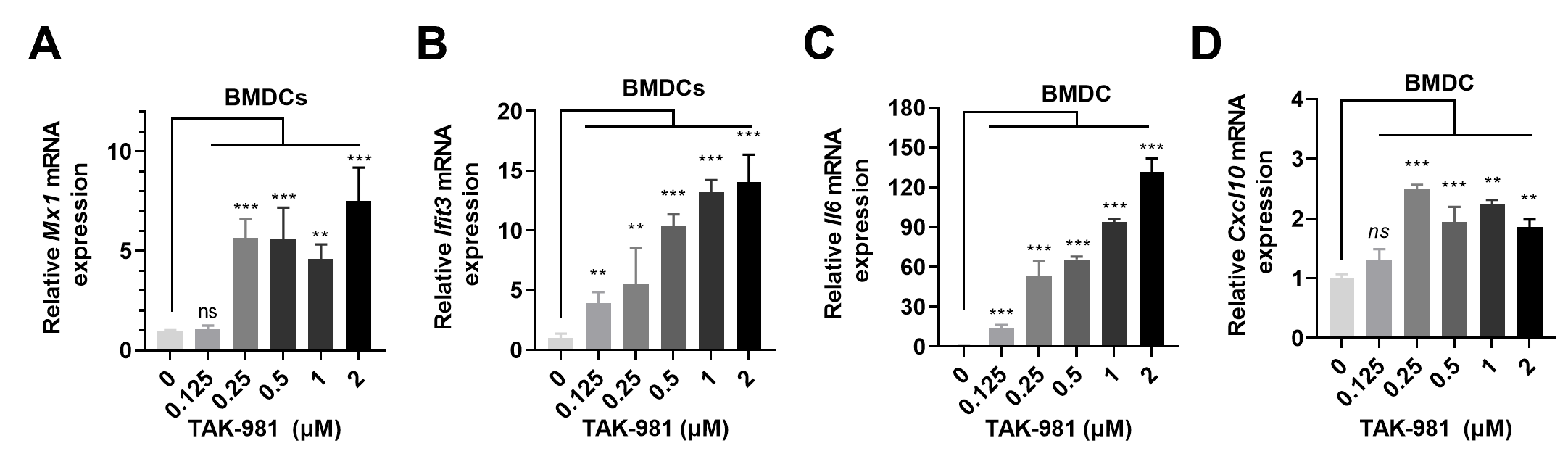


**Figure S10.** Relative expression of ISGs in BMDCs co-cultured with heated Hepa1-6 cells pro-treated with TAK-981. ns, not significant, **p* < 0.05, ***p* < 0.01, ****p* < 0.001.

**Figure S11.** The hydrodynamic diameter distribution of the PLEL micelle.

**
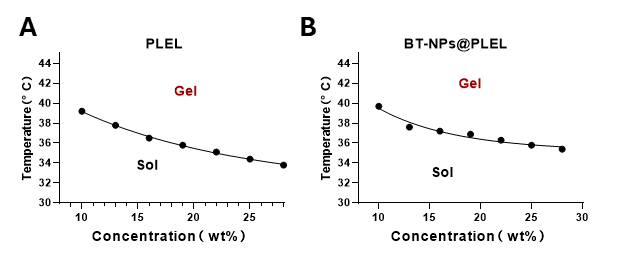
**

**Figure S12.** Concentration-dependent sol-gel transition properties of PLEL and BT-NPs@PLEL.

**Figure S13.** Cumulative release rate of TAK-981 from BT-NPs@PLEL for 14 days in 37° C (n = 6).


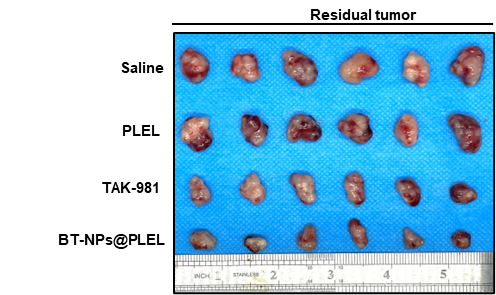


**Figure S14.** Representative images of residual tumors from different treatment groups on day21.

**
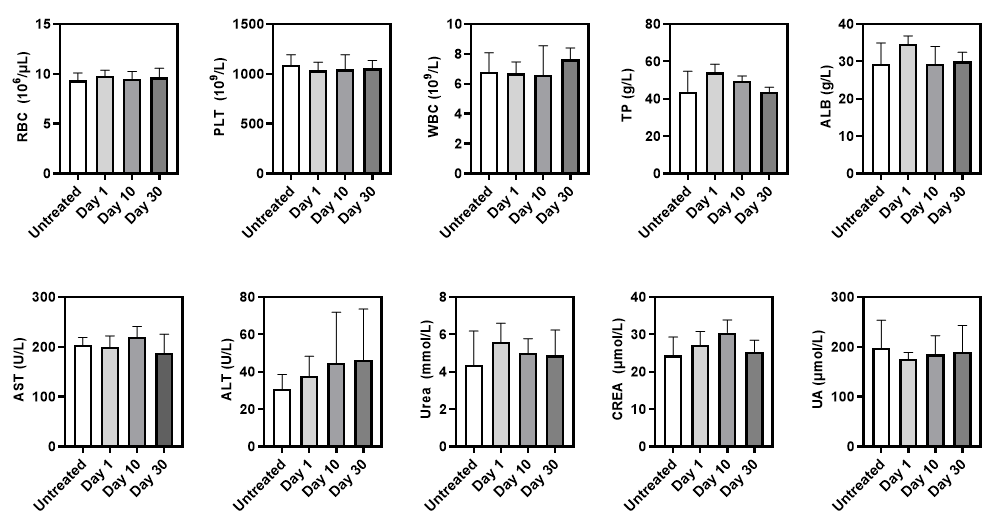
**

**Figure S15** Blood analysis of red blood cell (RBC), white blood cell (WBC), platelets (PLT), total protein (TP), albumin (ALB), Aspartate aminotransferase (AST), alanine aminotransferase (ALT), urea nitrogen (Urea), creatinine (Crea), uric acid (UA) levels after treatment with BT-NPs@PLEL on day 1, day 10, day 30.

**
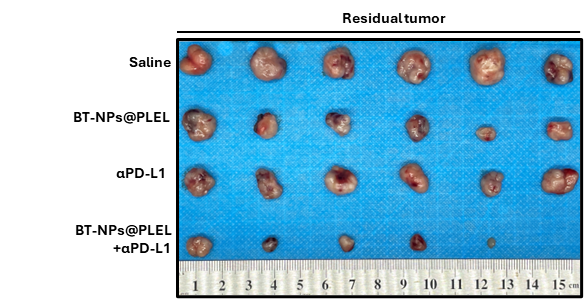
**

**Figure S16.** Representative photograph of residual tumors post–iRFA after different treatment on day 21.

**
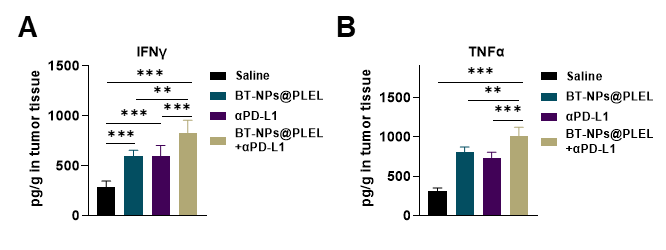
**

**Figure S17**. ELISA assay to detect the amount of IFN-γ, TNF-α in residual tumors after different treatment (n =6). ns, not significant, **p* < 0.05, ***p* < 0.01, ****p* < 0.001.


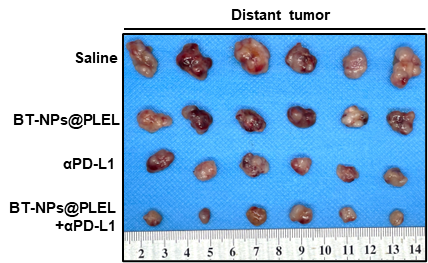


**Figure S18.** Representative photograph of distant tumors on day 21.


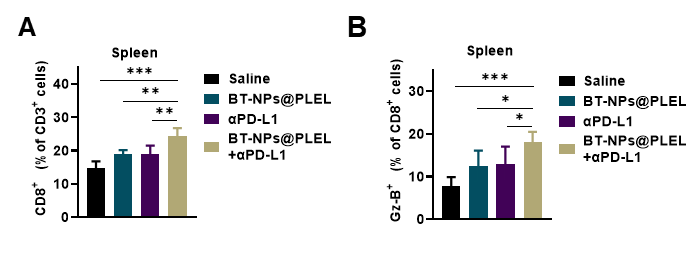


**Figure S19.** The proportions of CD8^+^ T cells and Granzyme B^+^ cells of CD8^+^ T cells of in spleen after different treatment(n=6). **p* < 0.05, ***p* < 0.01, ****p* < 0.001.


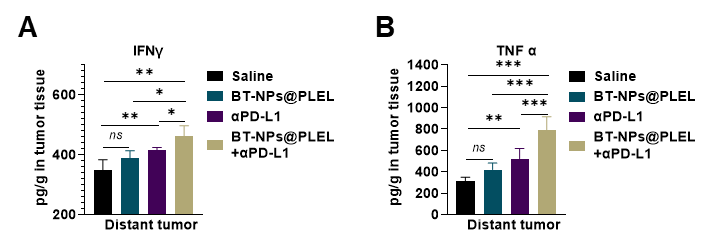


**Figure S20.**ELISA assay to detect the amount of IFN-γ, TNF-α in distant tumor tissue (n =6). ns, not significant, **p* < 0.05, ***p* < 0.01, ****p* < 0.001.

**
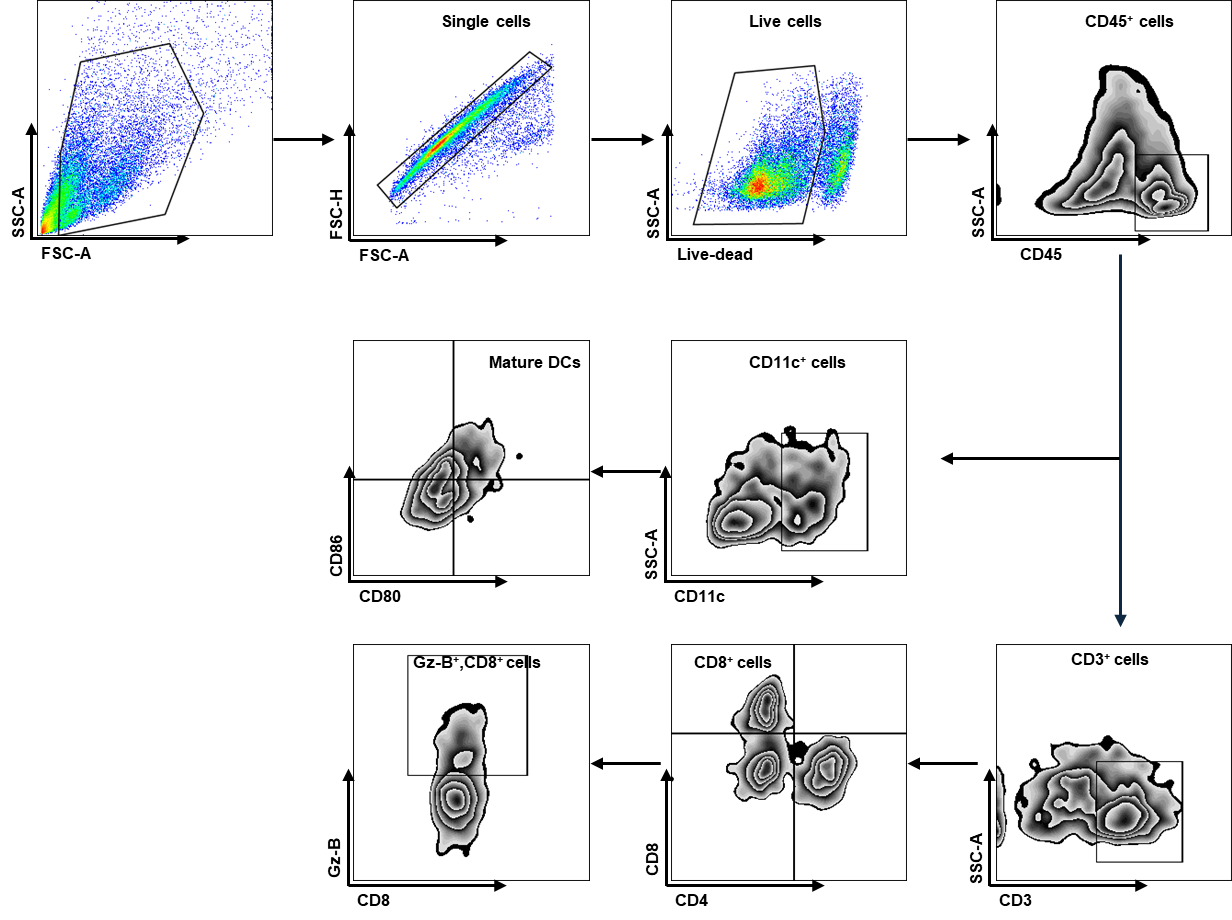
**

**Figure S21.** Gating strategy of DCs and T cells in tumors.
